# Rescue of ciliogenesis and hyperglutamylation mutant phenotype in *AGBL5*^−/−^ cell model of retinitis pigmentosa

**DOI:** 10.1186/s12860-025-00551-x

**Published:** 2025-09-09

**Authors:** Suly S. Villa-Vasquez, Liliya Nazlamova, Reuben J. Pengelly, David I. Wilson, Diana Baralle, Gabrielle Wheway

**Affiliations:** School of Human Development and Health, Faculty of Medicine, University of Southampton, Southampton, UK

**Keywords:** Cilia, Ciliopathies, Retinitis pigmentosa, Glutamylation

## Abstract

Retinitis pigmentosa (RP) affects around 1 in 4000 individuals and represents approximately 25% of cases of vision loss in adults, through death of retinal rod and cone photoreceptor cells. It remains a largely untreatable disease, and research is needed to identify potential targets for therapy. Mutations in 94 different genes have been identified as causing RP, including *AGBL5* which encodes the main deglutamylase that regulates and maintains functional levels of cilia tubulin glutamylation, which is essential to initiate ciliogenesis, maintain cilia stability and motility. In this study we use CRISPR-mutated *AGBL5* clonal retinal pigmented epithelial cell lines to characterise the cilia defects and hyperglutamylation in these cells and identify potential targets for treatment. We demonstrate rescue of glutamylation to wild-type levels and restoration of ciliogenesis in *AGBL5* mutant cells through exogenous expression of *AGBL5*, and independently through both stable genomic mutation and transient siRNA knockdown of *TTLL5*, which encodes a tubulin glutamylase. This identifies two potential routes to treatment for patients with RP associated with mutations in *AGBL5* which will need to be explored further in retinal organoid models of this disease.

**Supplementary Information:**

The online version contains supplementary material available at 10.1186/s12860-025-00551-x.

## Introduction

Retinal ciliopathies are a group of diseases caused by ciliary defects in cells of the retina, including the rod and/or cone photoreceptors, and retinal pigment epithelium (RPE). Typically, defects in cilia of these cells lead to retinal degeneration and visual impairment, which may be present as a feature of multisystemic ciliopathies, but also exist as non-syndromic or isolated retinal ciliopathies, including retinitis pigmentosa (RP), macular degeneration, cone-dystrophy, cone-rod dystrophy and Leber congenital amaurosis, with RP being the most common retinal manifestation of a ciliary defect [1–3].

One of the genes associated with retinal ciliopathies in humans is ATP/GTP Binding Protein Like 5 (*AGBL5*; OMIM 615900; Ensembl ID ENSG00000084693), also known as Cytosolic Carboxypeptidase-Like Protein 5 (*CCP5*). Human *AGBL5* is a 16-exon, 26 kb protein coding gene located on chromosome 2 (NC_000002.12) 2p23.3. The gene encodes 2 main protein-coding transcript variants; NM_021831.6 is a 3188 bp transcript of 15 exons encoding an 886 amino acids protein of ~ 97.5 kDa, which has been described as the canonical variant, and NM_001035507.3, a 2382 bp transcript of 11 exons which encodes a 717 amino acids protein of ~ 80 kDa.

*AGBL5* encodes a metallo-carboxypeptidase which catalyses the hydrolysis of C-terminal amino acids, and is member of the M14 family protein, which means that its active site contains a catalytic zinc [4]. This subfamily of Cytosolic Carboxypeptidase-Like proteins was identified in 2007 and named as CCP1 through 6 [4, 5]. Initial studies in mice showed that CCP5 is particularly abundant in testis, moderate in pituitary, brain, eye, and kidney and lower in other tissues including muscle, heart, lung, pancreas, intestines, liver, and spleen. At the cellular level, CCP5 was found to be distributed in the cytosol with slight enrichment in the nucleus [5] and in cilia [6].

In 2013, CCP5 was described as a dual-functional enzyme that removes glutamate residues from side chains of α- and β-tubulin, and C-terminus of α-tubulin, although it preferentially removes branching points generated by glutamylation, thus allowing the complete reversal of the polyglutamylation modification [7, 8]. Subsequently, a study in zebrafish revealed ccp5 as the main deglutamylase that regulates and maintains functional levels of cilial tubulin glutamylation, which is essential to initiate ciliogenesis, maintain cilia stability and motility [9]. Even though tubulin is its main substrate, CCP5 has been shown to act on non-tubulin substrate cGAS [10]. NAP1 and RPGR have been identified as potential substrates of CCP5 [11, 12].

Studies also showed that zebrafish *ccp5* morphants exhibit cilia tubulin hyperglutamylation, which caused uncoordinated beating and even complete paralysis of individual cilia; in addition, *ccp5* knockdown induced ciliopathy phenotypes such as hydrocephalus, axis curvature and renal cysts. Knockdown of other *ccps* such as *ccp1*,* 2* and *6* did not induce ciliopathy phenotype [9, 13]. In mice lacking Ccp5, spermatogenesis is defective, with ectopic tubulin polyglutamylation in developing sperm cells leading to mature sperm with abnormal morphology [14]. Overduplication of centrioles was found in spermatids, as well as assembly or multiple but defective axonemes, suggesting that glutamylation could control centriole duplication. Furthermore, no functional sperm were formed. Evidence suggests that hyperglutamylation resulting from loss of Ccp5 is associated with morphological defects during murine spermatogenesis that lead to infertility [15]. More recently, two studies have shown that *Ccp5*^*−/−*^ mice undergo retinal degeneration with hyperglutamylation of tubulin in the photoreceptor cells [16, 17]. In one study, hyperglutamylation was shown to be associated with shortened photoreceptor axonemes and the formation of numerous abnormal membranous whorls that disrupted the integrity of photoreceptor outer segments (OS) [16]. In the other study, hyperglutamylation in *Ccp5*^*−/−*^ mice retina was shown to be associated with extension of the connecting cilium (defined by POC5 localisation) and loss of the bulge region (defined by LCA5 localisation) where the membrane discs are formed, and mislocalisation of cilia proteins such as intraflagellar transport proteins [17]. Collectively these studies suggests that Ccp5 is important for normal balance of glutamylation of tubulin in the photoreceptors, and associated cell morphology, particularly at the connecting cilium and bulge region where membrane discs are formed [16, 17].

The first report of AGBL5-associated disease in humans was published in 2015, it described an exome sequencing study in five Turkish families with RP and identified a homozygous missense mutation in the zinc finger domain of *AGBL5*, a highly conserved region among the CCPs and in orthologs. The homozygous mutation was present in all affected individuals and the unaffected parents were heterozygous for this mutation. *AGBL5* was proposed as a candidate gene for human ciliopathies. Human and mouse retina were found to express AGBL5 in all layers, and a 97 kDa band was detected by western blot in retina, brain, and testis. cDNA analysis in normal human retina showed a potential splice variant missing exon 2, whilst in mouse retina a splice variant missing exon 3 was found [18]. Since this report 5 more studies have described 11 missense and frameshift mutations in patients with non-syndromic retinitis pigmentosa [19–23].

As a gene recently discovered to be involved in human retinal disease, we investigated AGBL5 function in a convenient and well-characterised human retinal pigmented epithelial cell line, in order to expand the understanding of the role of *AGBL5*, validate molecular insights obtained from studies of the *Ccp5*^*−/−*^ mouse model in a human model, and provide a useful and easy to manipulate model with which to test the molecular effect of potential therapeutics.

## Materials and methods

### Cell lines and culture

Adult retinal pigmented epithelial (ARPE19) wild-type (WT) and *AGBL5*^*−/−*^ clones A7 and A9 were obtained from Synthego Corp. Cells were cultured in Dulbecco’s Modified Eagle Medium/Nutrient Mixture F-12 (DMEM/F12 1:1 mix, Gibco) supplemented with 10% New-born Calf Serum (NBCS, Sigma), and incubated at 37 °C, in humid atmosphere of 5% CO_2_.

### Genotyping of ARPE19 WT and AGBL5^-/-^ clones

Primer sequences to amplify ARPE19 *AGBL5* WT and *AGBL5*^*−/−*^ clones’ CRISPR mutation can be found in Supplementary Table S1 . PCRs were performed using OneTaq^®^ 2X Master Mix with Standard Buffer (NEB), with genomic DNA as template. Sanger sequencing was performed using the forward primer, by Source Biosciences.

### Off-target analysis

Cas-OFFinder tool (http://www.rgenome.net/cas-offinder/) was used to search for off-targets using the single guide RNA sequence provided by Synthego TCTCGAATGCGTTCCCAGCG (PAM *NGG*). The search options included CRISPR/Cas9-derived RNA-guide Endonuclease: SpCas9 from *Streptococcus pyogenes*: 5’-NGG-3’, mismatch number equal or less than 4, DNA bulge size equal or less than 2, and RNA bulge size equal or less than 2. The results were analysed using BLAST to identify the nature of the region (exon, intron, intergenic region, lnRNA) and BAM files obtained from RNA-seq were visualised on the Integrative Genomics Viewer (IGV) to identify potential mutations.

### Generation and genotyping of AGBL5^-/-^/RET^-/-^ and AGBL5^-/-^/TTLL5^-/-^ pools

A pool of three guide RNAs obtained from Synthego were complexed together with spCas9 to form a ribonucleoprotein (RNP). RNPs were then delivered to the cells via nucleofection using the Nucleofector Lonza 4D X-unit and the SF Cell Line kit. 72 h after nucleofection, the cell pools were assessed for efficiency of edits using Sanger sequencing and the ICE CRISPR analysis tool (https://ice.synthego.com/).

### TTLL5 knockdown using SiRNA

*TTLL5* knockdown was performed ON-TARGETplus human TTLL5 siRNA SMARTpool from Dharmacon and lipofectamine RNAiMAX (ThermoFisher). A reverse transcription protocol was followed, and siRNA duplexes were added at a final concentration of 50nM per well. ON-TARGETplus human GAPD Control Pool and ON-TARGETplus Non-Targeting Control Pool (Dharmacon) were used as positive and negatives controls. Transfected cells were cultured in Opti-MEM I Reduced Serum Medium (Gibco) for 48 h for RNA extraction and qPCR, or 120 h for protein extraction, western blot and immunocytochemistry. Experiments were performed in triplicate for gene expression, and duplicates for western blot and immunocytochemistry.

### SiRNA knockdown confirmation by qPCR

RNA was extracted from cells using QIAGEN RNeasy mini kit and following the manufacturer’s instructions. cDNA was synthetised using the High-Capacity cDNA Reverse Transcription Kit (ThermoFisher), and qPCR was performed using PowerTrack SYBR Green Master Mix (ThermoFisher). 10ng of cDNA were used per qPCR reaction and run on the StepOnePlus Real-Time PCR System (ThermoFisher) standard run with melt curve. Quantification of gene expression was performed using the ∆∆C_T_ method and normalised to actin-beta (*ACTB*) gene levels. The primers can be found in Supplementary Table S1 .

### RNA extraction, purification and quality control

RNA was extracted from cells using TRIzol reagent. Contaminating DNA was removed using the TURBO DNA-free™ Kit (ThermoFisher) following the manufacturer’s instructions. The sample volume was adjusted to 50 µl using nuclease-free water, and additional cleaning and concentration steps were performed using the RNA Clean & Concentrator^TM^-5 kit (Zymo Research) following manufacturer’s instructions. RNA was eluted in 11 µl of DNase/RNase-Free water. RNA quality was measured using an RNA Nano chip on the Agilent Bioanalyser 2100. RNA samples with RNA integrity number (RIN) over 9.0 were taken forward for sequencing.

### cDNA library preparation and sequencing

cDNA libraries were prepared using NEB Next^®^ Ultra™ RNA Library Prep Kit by Novogene Co, which generates a non-stranded library. Library size distribution was assessed using Agilent Bioanalyser 2100 and quantification was checked using Qubit and real-time PCR. Libraries were pooled for paired-end 150 bp sequencing to a depth of 50 million reads per sample, on an Illumina NovaSeq 6000 system by Novogene Co.

### RNA-seq analysis

Raw FASTQ reads were filtered by Novogene Co. to remove reads containing adapters, reads containing *N* > 10%, and reads with low quality (Qscore ≤ 5) in bases over 50% of the read. These FASTQ files are deposited on European Nucleotide Archive and are accessible under accession numbers PRJEB96053 (Project) and ERP178802 (Study). Quality of the filtered sequence data was assessed using FastQC v0.11.3 (https://www.bioinformatics.babraham.ac.uk/projects/fastqc/), and no additional data filtering was required.

Paired FASTQ files were aligned to the GRCh38 human genome reference using STAR v2.7.5a splice aware aligner [24], and basic options of the two-pass alignment method, with soft clipping activated. BAM files were sorted by chromosomal coordinates and enquired for saturation of known splice junctions using RSeqQC v4.0.0 [25] versus the Gencode v38 BED file. Reads mapped to Gene ID were counted using HTSeq v0.6.1 [26] and Gencode v38 annotations [27], and differential gene expression analysis was performed with EdgeR. BAM files were visualized using IGV, and IGV was used to generate Sashimi plots. Transcript quantification was performed using Salmon v0.7.2 [28] and Gencode v41 human transcript annotations.

Gene ontology (GO) enrichment analysis was conducted on the Gene Ontology Consortium website (http://geneontology.org/, accessed in August 2024) [29, 30] powered by PANTHER [31]. The data was enquired for enrichment of GO terms related to biological processes using the PANTHER release 19 (released on 17th June 2024), Fisher’s Exact test with False Discovery Rate correction. GO results were sorted by fold enrichment and the top 25 GO terms were plotted using the SRplot platform [32] (https://www.bioinformatics.com.cn/srplot, accessed in August 2024).

### Verification of expression construct sequence

Primers to sequence the full length of the *AGBL5* NM_021831.6 ORF insert in the pcDNA3.1 + C-eGFP vector (GenScript) were designed using Primer3 software (https://primer3.ut.ee/). Sequencing primers were designed with 400 bp spacing and can be found in Supplementary Table S1 .

The sequencing results obtained for the *AGBL5* expression construct, revealed over 4550 bp of the sequence containing the CMV promoter, T7 promoter, MCS, *AGBL5* ORF, and eGFP tag. The sequencing allowed confirmation of the *AGBL5* insert sequence, the lack of stop codon and the absence of additional mutation.

### Transfection

AGBL5-eGFP expression construct was transfected into ARPE19 WT, *AGBL5*^−/−^ Clone A7 and Clone A9 using lipofection. 1 × 10^5^ cells were seeded on 24-well plates or 4 × 10^5^ cells on 6-well plates on sterile coverslips for rescue experiment. Media was replaced 6 h post-transfection and cells were serum starved for a total of 72 h to induce ciliogenesis. In all cases, the transfection with the plasmids of interest was performed alongside with the pmaxGFP control plasmid (Lonza).

### Protein electrophoresis and western blotting

Total proteins were mixed with equal volume of 2X SDS loading buffer and loaded onto precast NuPAGE™ 4–12%, Bis-Tris gel (ThermoFisher) alongside SpectraTM Multicolour Broad Range Protein Ladder (ThermoFisher). Proteins were separated by electrophoresis at 200 V for 35 min using 1X MES SDS running buffer. Proteins were transferred to a PVDF membrane (ThermoFisher) at 35 V for 2 h with 1X transfer buffer. Membranes were blocked with a 5% non-fat milk in Phosphate Buffered Saline PBS (w/v) blocking solution for 1 h at room temperature in agitation and incubated with primary antibody overnight at 4 °C or for 1 h at room temperature in constant agitation. The blots were visualised using SuperSignal West Femto substrate (ThermoFisher) and chemiluminescence settings in an iBright 15,000 imaging system (ThermoFisher). Densitometry analysis was performed using the iBright Analysis Software. Details of the primary and secondary antibodies can be found in Supplementary Tables S2 and S3.

### Immunofluorescence staining

Cells grown on glass coverslips were fixed and cilia were stained using antibodies against glutamylated tubulin (GT335, Adipogen AG-20B-0020) 1:500, and the cilia membrane marker ARL13B (Proteintech 17711-1-AP) 1:200. Transfection with the *AGBL5* expression construct was detected by the expression of the eGFP tag. Nuclei were stained with DAPI. Details of the primary and secondary antibodies can be found in Supplementary Tables S2 and S3.

### Confocal microscopy

Manual confocal image capture was performed using a Leica SP5 confocal laser scanning microscope attached to a Leica DMI6000 inverted epifluorescence microscope, using a HCX PL APO CS 63.0x/1.30 GLYC 21 °C UV glycerol immersion objective, 405, 488, and 561 nm lasers. Images were captured using the LAS AF software and the pinhole set at 1 AU. Images were exported as .lif files and TIFF for manual analysis on ImageJ [33].

### High resolution confocal imaging – hyvolution

Confocal images were obtained by the Wolfson Bioimaging Centre staff at the University of Bristol. Imaging was carried out on a Leica SP8 AOBS confocal laser scanning microscope attached to a Leica DMi8 inverted epifluorescence microscope, using a HC PL APO 63x/1.40 oil objective, 405 nm, 488 nm, 561 nm, and 633 nm lasers, one standard PMTs and two high sensitivity hybrid HyD SMD detectors. Images were captured using LASX software with Hyvolution 2, and pinhole set at 0.5 AU. Images were deconvolved using automated Huygens Essential Maximum Likelihood Estimation (MLE) developed by SVI (Scientific Volume Imaging), with standard strategy. Images were exported as .lif files and TIFF for manual analysis on ImageJ.

### Spinning disk confocal super resolution imaging – SpinSR

Super Resolution confocal images were obtained by the Wolfson Bioimaging Centre staff at the University of Bristol, on an Olympus IXplore spinning disk system based on Yokogawa CSU-W1 SoRa (50 μm and SoRa disks), using a 60x/1.5 oil immersion objective, 405, 488 and 561 nm lasers. Images were captured using Olympus CellSens imaging software with 3D deconvolution and super-resolution modules, pinhole set at 1 AU. Images were exported as .vsi files for manual analysis on ImageJ.

### GFP-trap and mass spectrometry

GFP-Trap^®^ agarose beads (ChromoTek) were used to precipitate GFP tagged proteins from transfected cell lysates, following the manufacturer’s protocol. Liquid Chromatography tandem MS (LC-MS/MS) was performed at the mass spectrometry facility of the Institute of Genetics and Cancer, University of Edinburgh. On-bead digestion was performed, and the samples were run in a high-end machine Orbitrap Fusion Lumos (Tribid) Mass Spectrophotometer (Thermo Scientific). The data was acquired on DDA mode. MSFragger was used as database search tool for peptide identification (Human Proteome database: UniProtKB- Proteome ID: UP000005640; https://www.uniprot.org/proteomes/UP000005640). Statistical analysis was performed using an inhouse Limma Package R script.

### Coimmunoprecipitation (co-IP) of endogenous protein

500 µg protein lysates were incubated with 0.5 µg primary antibody and incubated at 4 °C for 2 h, in end-over-end rotation to allow binding of the antibody to the bait protein. 15 µl of a 50% Protein G Plus/Protein A Agarose Beads Suspension (Sigma) were equilibrated once with PBS and twice with wash buffer (10 mM Tris-HCl pH 7.5, 150 mM NaCl, 5 mM EDTA). Equilibrated beads were added to the antibody/protein lysate in end-over-end rotation overnight at 4 °C. The beads were sedimented by centrifugation at 1000 rcf for 1 min at 4 °C, washed three times using wash buffer, and resuspended in PBS. For elution, beads were boiled in equal volumes of 2X SDS sample buffer at 95 °C for 5 min.

### Statistical analysis

Normality of rescue experiments results was tested using the Shapiro-Wilk test. Two-way ANOVA and Bonferroni tests were used to calculate statistical significance when data was normally distributed; Kruskal–Wallis and Dunn’s tests were used for not normally distributed data. Analysis and graph construction were performed using GraphPad Prism 10.0.2.

## Results

### Characterisation of AGBL5^-/-^ mutant cell lines

We used a CRISPR/Cas9 knockout (KO) cellular model (ARPE-19 *AGBL5*^*−/−*^ cells) to study the functional role of *AGBL5* in human retinal pigmented epithelial cell lines. This line was produced by Synthego Corp, using SpCas9 from *Streptococcus pyogenes* and a sgRNA (TCTCGAATGCGTTCCCAGCG (PAM *NGG*)) targeting exon 3, an exon common to both *AGBL5* protein-coding transcripts. Cells were clonally isolated using single cell dilution, and clones grown up. We studied two clonal lines; A7 and A9 from passage 13 onwards, and a passage 8 wild-type (WT) matched unedited cell line.

**Fig. 1:**
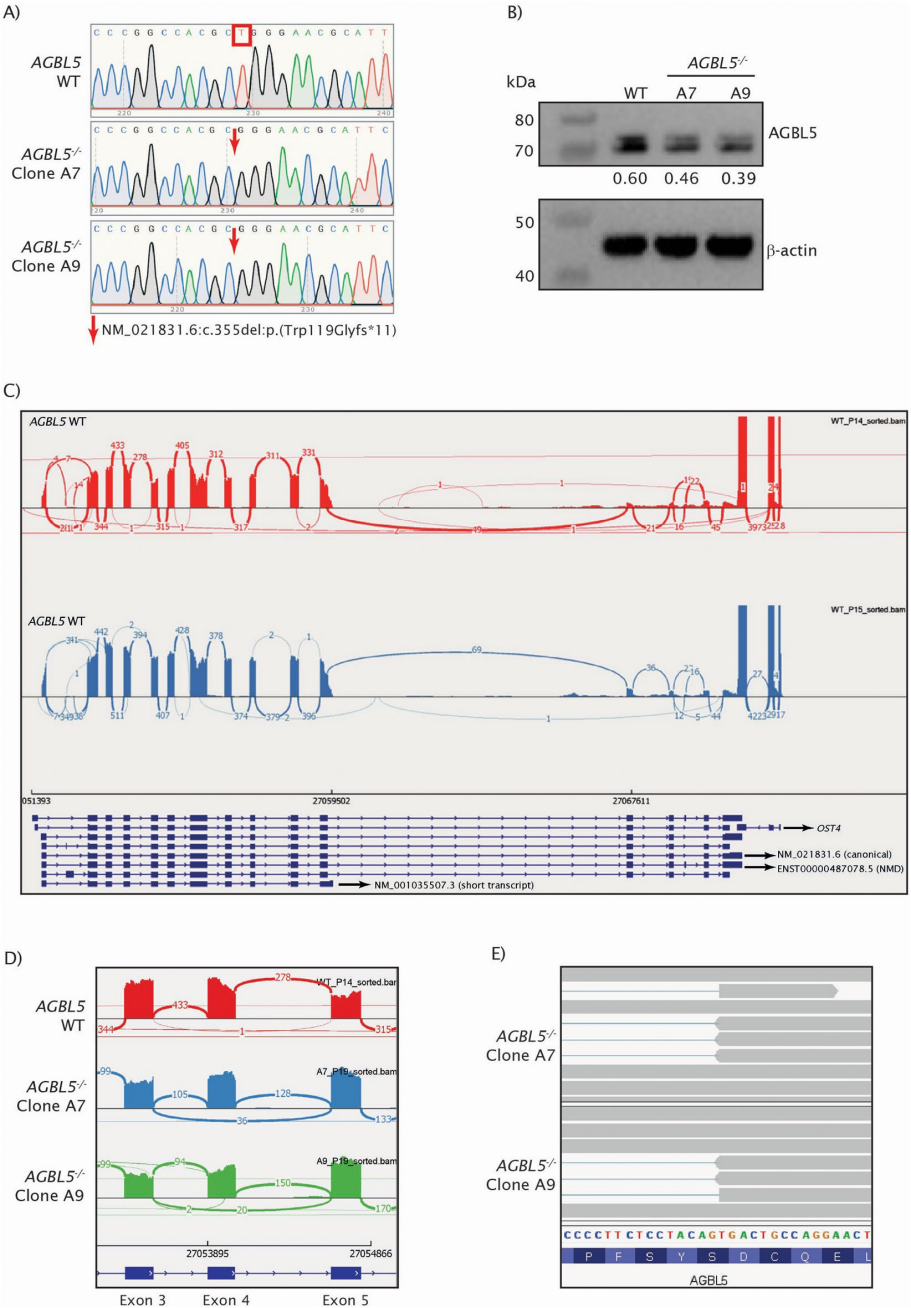
Characterisation of genetic changes in the *AGBL5*^*−/−*^ CRISPR mutant cell line, and the effect of those changes on *AGBL5* RNA expression. **a**) Sanger trace of the edited region in ARPE19 *AGBL5* WT, clone A7 and clone A9, showing 1 bp indel in exon 3 (arrows). **b**) Western blot showing AGBL5, loading control (β-actin) in ARPE19 *AGBL5* WT, clone A7 and clone A9, and densitometry values of AGBL5 normalised to the loading control. **c**) Sashimi plot showing exon skipping of exon 4 in *AGBL5*^*−/−*^ clones. **d**) IGV screen capture showing usage of an alternative splice site in exon 4 in *AGBL5*^*−/−*^ clones. **e**) IGV screen capture showing usage of an alternative splice site in exon 4 in *AGBL5*^*−/−*^ clones. Note: this same sequence can be observed in the Sanger sequencing traces presented in Supplementary Figure S1 (panel B)

We confirmed presence of a homozygous 1 bp deletion in exon 3 (GRCh38 chr2:27,053,541, NM_021831.6:c.355del) of the *AGBL5*^−/−^ clones A7 and A9, compared to the WT cells (Fig. 1a). This deletion occurs in exon 3, a highly conserved region of this gene. The deletion replaces a tryptophan (Trp) in position 119 with a glycine (Gly) and introduces an early stop codon 11 amino acids away from the mutation site. If translated, the translation would be expected to stop in the exon and the predicted translated product would be a 129 amino acids protein of 14.09 kDa. The search for off-targets revealed potential off-targets in 14 genomic locations when DNA and RNA bulge sizes were set at zero. The targets are intronic regions of 8 genes, 3 long noncoding RNAs and 3 intergenic regions. The potential off-target effects at these sites are unlikely to have functional effects. When DNA and RNA bulge options were expanded, 337 additional sites appeared. All 351 predicted off-target sites were visualized in BAM files from total RNA seq data from the mutant clones in IGV to assess the presence of mutations, however, no sequence changes were identified in any of the regions covered by RNA-seq.

To confirm loss of both full-length AGBL5 protein isoforms we carried out western blotting of lysates from the WT and mutant cells, and unexpectedly we observed bands of approximately 70–75 kDa, and a slight (~ 40%) reduction in the intensity of these bands in the mutant cells compared to WT cells, which was reproducible over several repeats (*n* = 9) (Fig. 1b, Supplementary Figure S1). The presence of these double bands as well as the difference in the expected molecular size versus the size observed could be explained by protein cleavage and/or post-translational modifications, or the presence of different splice variants. The reduction in AGBL5 band intensity in *AGBL5*^−/−^ clones A7 and A9, compared to the WT cells was reproducible using two different antibodies for AGBL5 (NBP1-56622 and NBP3-10627). To investigate the possibility of the presence of novel splice variants further, we obtained RNA from the WT and *AGBL5*^−/−^ cells, converted to cDNA and used various primer combinations (Supplementary Table S1) to attempt to amplify the different AGBL5 transcripts present in the cells. We obtained PCR products of various sizes, extracted and purified each band from the gel and sequenced it using Sanger sequencing. This showed one transcript present in WT cells, including the canonical exon 3 and exon 4. In the *AGBL5*^−/−^ cells we observed a number of splice variants including exon 3 spliced to a partial exon 4, partial exon 2 spliced to a partial exon 8, partial exon 2 spliced to a partial exon 9 and partial exon 3 spliced to intron 3 (Supplementary Figure S2). To validate these findings, we carried out total (rRNA depleted) mRNA sequencing of all cell lines (WT *n* = 2, *AGBL5*^−/−^ A7 *n* = 2, and *AGBL5*^−/−^ A9 *n* = 3). This revealed that a number of *AGBL5* transcripts are expressed in ARPE19 cells (Fig. 1c), with NM_001035507.3 (2,382 bp 11-exon transcript), predicted to encode a ~ 80 kDa protein, the predominant transcript, and NM_021831.6 (3,188 bp 15-exon transcript), predicted to encode a ~ 97.5 kDa protein, expressed at a lower level (quantification of the expression of each *AGBL5* transcript in every sample analysed can be found in Supplementary Table S4). No additional splice variants were observed. RNAseq results therefore suggest that the bands observed by western blot originate from the short (11-exon) transcript, and that the molecular weight observed and the presence of double bands could be the result of post-translational modifications. Furthermore, a decrease in the RNA levels of AGBL5 was also observed in the mutant clones, which could lead to low production of a mutant version of the AGBL5 protein, potentially consistent with the bands of similar molecular weight but lower intensity obtained by western blot in the two mutants. This is different from the data presented in Kastner et al. 2015, which described a ~ 97.5k Da band in human tissues [18]. To the best of our knowledge, there are no other published examples of AGBL5 examined by western blot.

**Fig. 2:**
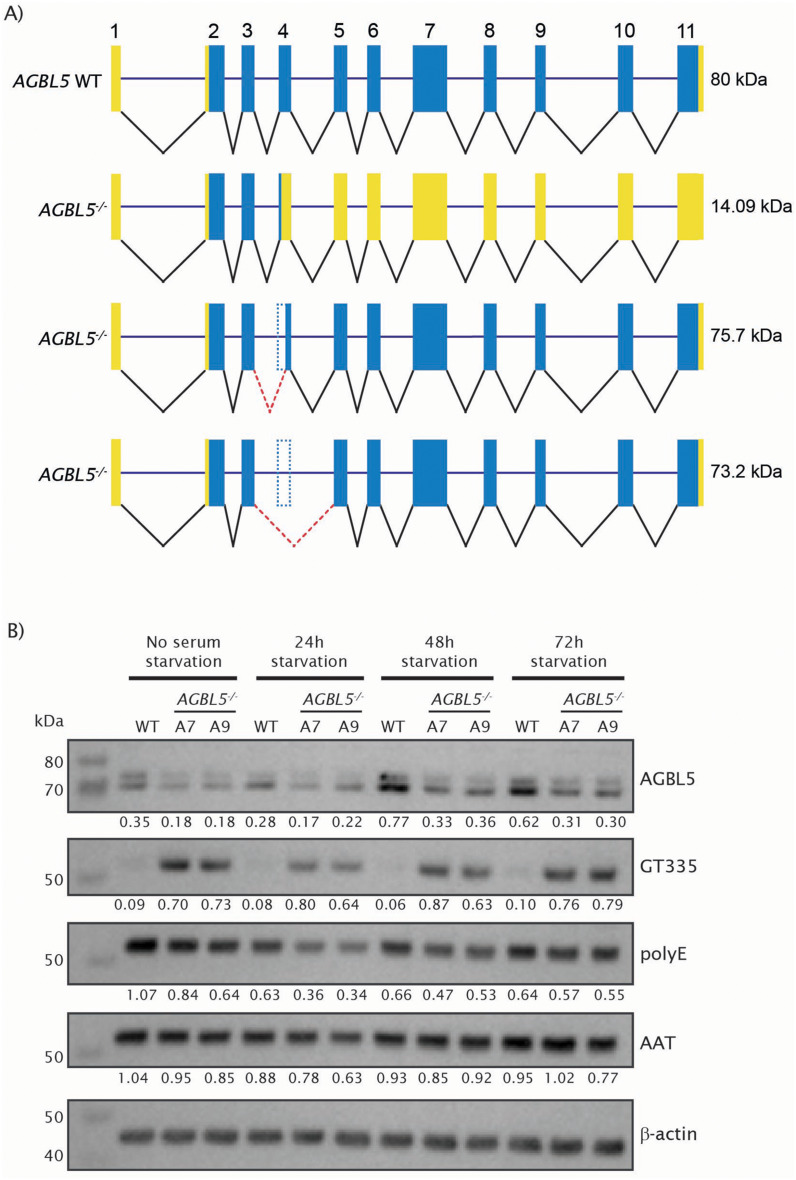
Characterisation of the effects of mutations in *AGBL5*^*−/−*^ mutant cell line on *AGBL5* splicing, AGBL5 protein expression, tubulin glutamylation and polyglutamylation. **a**) Schematic representation of WT AGBL5 short transcript splicing and alternative splicing of AGBL5 short transcript in *AGBL5*^*−/−*^ mutant cell line. **b**) Representative western blot showing expression of AGBL5, glutamylated tubulin GT335, polyglutamylation PolyE, acetylated α-tubulin and the β-actin loading control under growing conditions (10% NBCS) or serum starvation (1% NBCS). Densitometry values normalised to the loading control

Sashimi plots of the RNAseq data revealed usage of exons 3 and 4 in all cells, and partial and total exon skipping of exon 4 in *AGBL5*^−/−^ clones A7 and A9 (Fig. 1d), avoiding the stop codon resulting from the CRISPR frameshift mutation. Skipping of exon 4 retains the reading frame in the transcript, leading to a protein with a loss of 55 amino acids. The RNAseq data also showed use of an alternative splice site in exon 4 which led to deletion of 98 bp of this exon (Fig. 1e), which removes the stop codon introduced by the frameshift mutation and allows the protein to continue its translation until the stop codon in exon 11. The splicing changes observed in the RNAseq data were confirmed by Sanger sequencing of RT-PCR products from the *AGBL5*^−/−^ cells. Translation of these altered transcripts would result in proteins with theoretical molecular weights of 75.7 kDa when partial exon 4 skipping occurs (98 bp deletion), and 73.2 kDa for total exon 4 skipping, corresponding with the sizes of the bands seen on the western blot. These results suggest that complex aberrant splicing events are taking place in the *AGBL5*^−/−^ cells. A schematic representation of these splicing events in the short (11-exon) *AGBL5* transcript is presented in Fig. 2a.

We proceeded to investigate whether the full or partial skipping of exon 4 had a functional effect on tubulin glutamylation levels in cells, as AGBL5 is a known tubulin deglutamylase. First, we extracted total proteins from WT and *AGBL5*^−/−^ clones at different stages of serum starvation and observed levels of tubulin glutamylation (GT335) in *AGBL5*^−/−^ clones much higher than in WT cells. This indeed suggests that the *AGBL5*^−/−^ clones produce defective AGBL5 which cannot remove the branching point glutamate (Fig. 2a). This is consistent with previous studies which have shown that AGBL5 is a major deglutamylase which removes glutamate at the branch point [49, 58]. However, the levels of polyglutamylation (polyE) were lower in *AGBL5*^−/−^ clones compared to WT (Fig. 2b). This is different from what we expected, as we anticipated to see no changes or possibly an increase in the polyglutamylation signals, resulting from the inability of AGBL5 to remove glutamate side-chains. These observations suggests that expression of other CCPs in ARPE19 cells, such as AGTPBP1, responsible for removal of side chain glutamates, may be regulating the length of the glutamate side chains. As PolyE can detect any glutamate side chain, not exclusively on tubulin, we focused on the bands ~ 50–55 kDa which overlapped with GT335 and with acetylated α-tubulin (AAT), but it is possible that there is some non-specificity in this result. The levels of AGBL5 protein were consistently slightly lower in *AGBL5*^−/−^ clones than in WT cells throughout all the conditions.

**Fig. 3:**
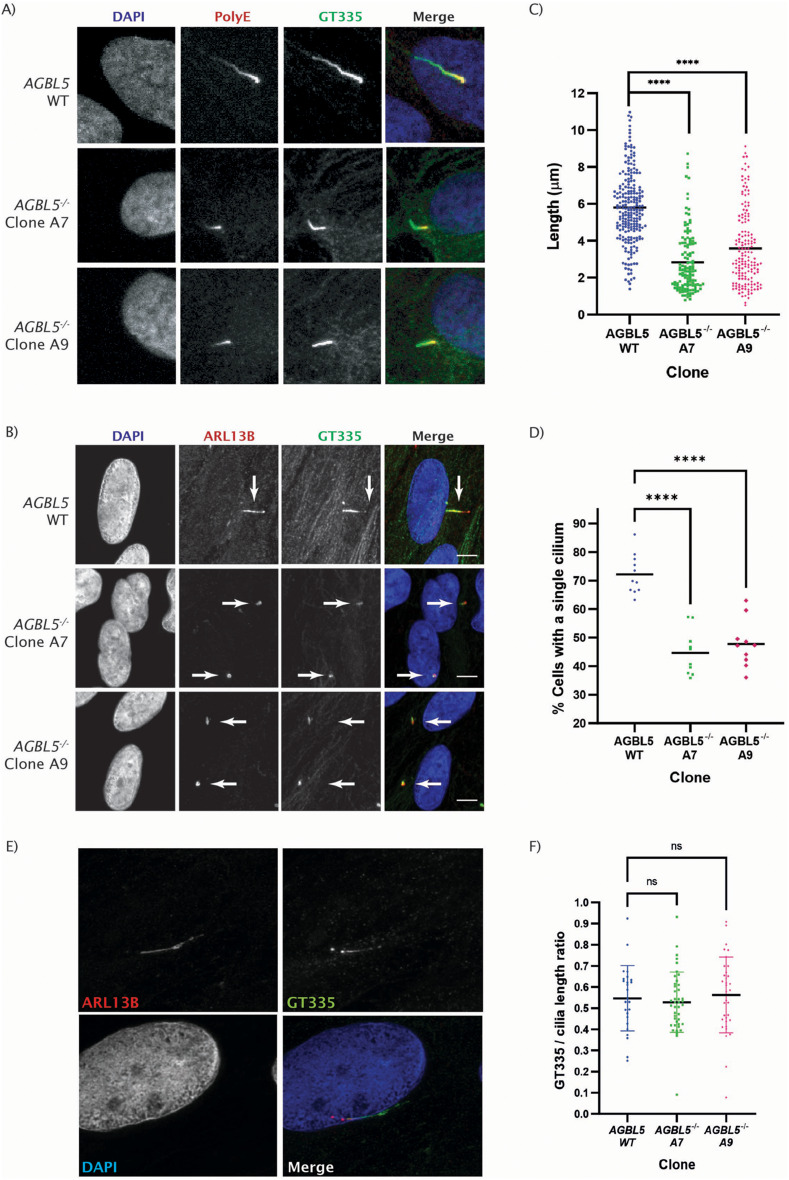
Characterisation of the cilia phenotype of *AGBL5*^*−/−*^ mutant cell lines. **a**) Representative immunocytochemistry images showing polyglutamylated tubulin stained with polyE antibody and glutamylated tubulin stained with GT335 antibody. **b**) Representative immunocytochemistry images showing cilia membrane staining (ARL13B) and glutamylated tubulin (GT335). Scale bar 10 μm. Arrows point to cilia. **c**) Dot plot from individually measured lengths of cilia. Bars represent the mean, **** *p* < 0.0001. **d**) Dot plot from individually measured percentage of cells with single cilium. Each dot represents a field of view. Bars represent the mean, **** *p* < 0.0001. **e**) Representative Hyvolution (proprietary Leica deconvolution method) image of cilia membrane staining (ARL13B) and glutamylated tubulin (GT335) along the cilium in a wild-type ARPE19 cell. **f**) Dot plot of the length of GT335 staining over the total cilia length showing no statistically significant difference (ns)

Next, we used confocal imaging using antibodies against glutamylated tubulin (GT335) and polyglutamylation (PolyE) to investigate further the changes in polyglutamylation, and the cilia membrane protein ARL13B to investigate the cilia phenotype. GT335 staining was observed in cytoplasmic microtubules and cilia of AGBL5 WT and *AGBL5*^*−/−*^ cells, and the fluorescence intensity was higher in *AGBL5*^*−/−*^ clones, particularly in those signals in the cytoplasm. On the other hand, polyglutamylation signals (PolyE) were enriched in the cilia, particularly towards the base of the cilium (Fig. 3a). The results also revealed the presence of shorter cilia in the *AGBL5*^−/−^ clones compared to the WT cells, cilia in *AGBL5*^−/−^ clone A7 were 51.4% shorter than cilia in WT cells, and cilia in *AGBL5*^−/−^ clone A9 were 38.3% shorter (Fig. 3b and c). Reduction in the number of ciliated cells was also observed in *AGBL5*^−/−^ cells, the percentages of ciliated cells were WT 70.2%, *AGBL5*^−/−^ clone A7 40.1% and *AGBL5*^−/−^ clone A9 46.94%, suggesting that loss of AGBL5 also disrupts ciliogenesis (Fig. 3d). This further suggests that the decrease in polyE signal in *AGBL5*^−/−^ cells observed by western blot potentially results from the presence or shorter and fewer cilia, rather than an increase in the number or length of glutamate side-chains. The staining with GT335 also allowed the identification of only 1 basal body per cell in all clones.

Hyvolution imaging allowed a closer assessment of the localisation of glutamylated tubulin along the cilium. The GT335/cilia length ratio was calculated as the length of GT335 staining divided by the length of ARL13B staining. The ratios ranged from 0.08 to 0.93, but the media of these values was similar for all the *AGBL5* clones, and no statistically significant difference was detected (Fig. 3e and f).

### Rescue of cilia defects in AGBL5^-/-^ mutant cell lines via exogenous expression of AGBL5-eGFP

**Fig. 4:**
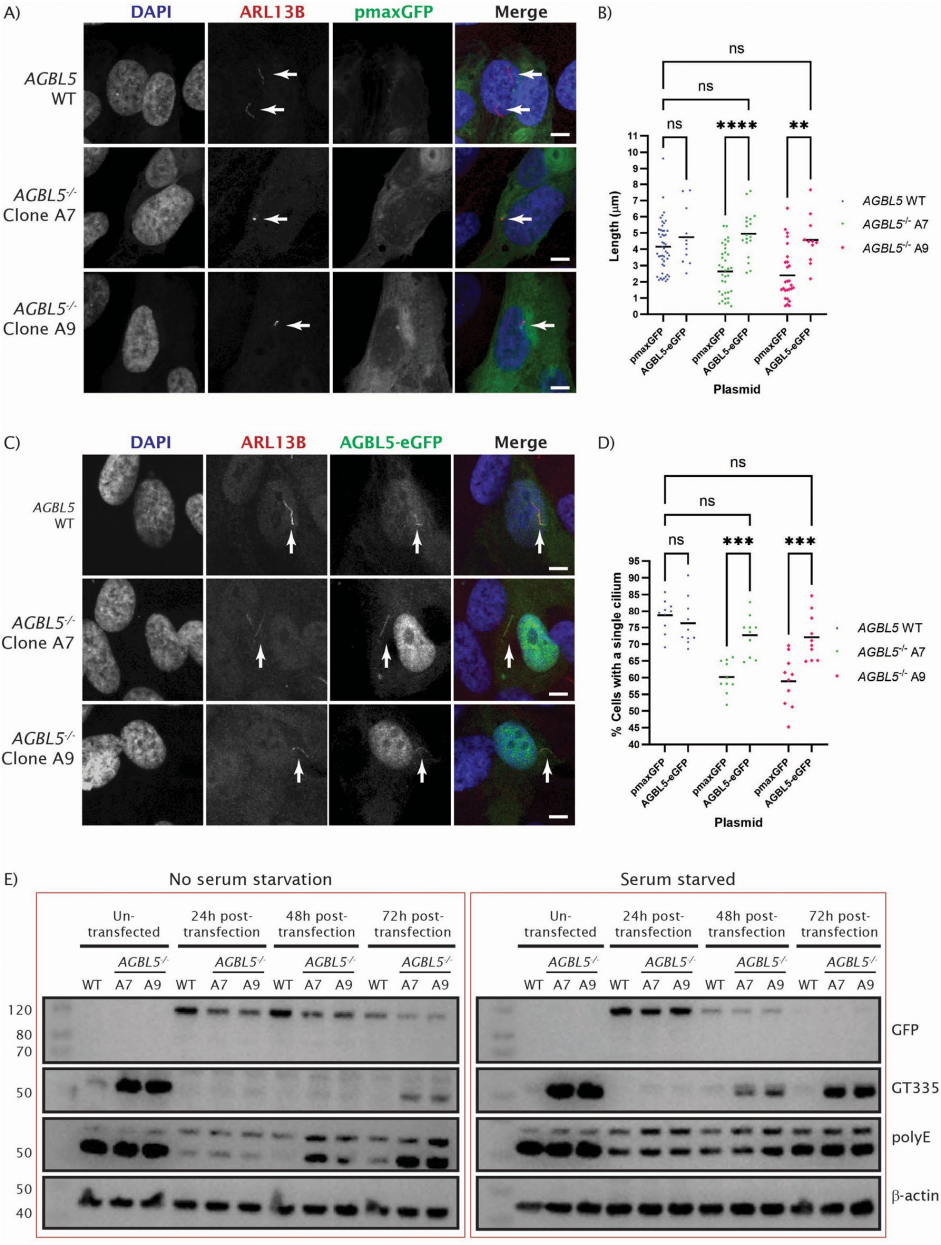
Rescue of ciliogenesis and hyperglutamylation in *AGBL5*^*−/−*^ mutant lines through exogenous *AGBL5* expression. **a**) Immunocytochemistry images showing lack of rescue of cilia defects in ARPE19 cells transfected with eGFP plasmid. Arrows point to cilia. Scale bar 10 μm. **b**) Dot plot of individual measures of cilia length, showing rescue in *AGBL5*^−/−^ clones transfected with AGBL5-eGFP plasmid expression. Bars represent the mean, **** *p* < 0.0001, ** *p* < 0.01, ns not significant. **c**) Immunocytochemistry images showing rescue of cilia defects in ARPE19 cells transfected with an AGBL5-eGFP plasmid expression. Arrows point to cilia. Scale bar 10 μm. **d**) Dot plot quantifying rescue of cilia number in *AGBL5*^−/−^ clones transfected with AGBL5-eGFP plasmid expression. Each dot represents a field of view. Bars represent the mean, *** *p* < 0.001, ns not significant. **e**) Western blot images showing expression of AGBL5, the GFP tag, glutamylated tubulin GT335, polyglutamylation PolyE, and the β-actin loading control (and showing rescue of hyperglutamylation in *AGBL5*^−/−^ clones) in ARPE19 cells transfected with AGBL5-eGFP plasmid expression. Left panel shows results for serum fed cells, right panel shows results when transfected cells are serum starved

To investigate the specificity of the cilia defects in the *AGBL5*^−/−^ mutant cell lines we attempted to rescue these phenotypes through exogenous expression of AGBL5-eGFP. We transfected empty pmaxGFP vector as a negative control. Analysis of cilium length in WT and the *AGBL5*^−/−^ clones when transfected only with GFP, revealed statistically significant shorter cilia in *AGBL5*^−/−^ clones, consistent with previous immunofluorescence experiments (Fig. 4a, b). However, cells transfected with the WT AGBL5-eGFP plasmid, showed a significant increase in the cilia length in *AGBL5*^−/−^ cells, with measurement comparable with that observed in WT cells (Fig. 4b, c). At this point, no statistically significant difference was detected between the *AGBL5*^−/−^ clones transfected with AGBL5-eGFP compared to the WT cells.

Analysis of the percentage of cells with a single cilium after transfection with GFP or WT AGBL5-eGFP, also showed significant increase of ciliogenesis in *AGBL5*^−/−^ cells transfected with AGBL5-eGFP, and no statistically significant difference when compared to the WT cells (Fig. 4d). These results suggest that transfection with the WT AGBL5-eGFP plasmid rescues the cilia phenotype in deficient cells. In addition, tagged AGBL5 protein can be seen localised in the cytoplasm, nucleus, and cilium.

We proceeded to investigate whether the hyperglutamylation seen in *AGBL5*^−/−^ clones was also rescued by AGBL5-eGFP expression. We transfected WT and the *AGBL5*^−/−^ clones with AGBL5-eGFP and extracted proteins 24, 48 and 72 h after transfection for analysis by western blotting. We observed AGBL5-eGFP expression, highest at 24 h post transfection, decreasing to 72 h after transfection. Interestingly, AGBL5-eGFP expression decreased more rapidly from 24 to 72 h post transfection in serum starved cells. With the AGBL5-eGFP expression we saw a concomitant decrease in glutamylation levels. This was most marked in the *AGBL5*^−/−^ clones, which showed very high levels of glutamylation (GT335) in untransfected cells, which was reduced to WT levels after exogenous expression of AGBL5-eGFP. Alongside this, we also observed a reduction in the levels of polyglutamylation (polyE) of all the *AGBL5* clones after AGBL5-eGFP expression (Fig. 4e).

The rescue of the ciliogenesis and glutamylation phenotypes in *AGBL5*^−/−^ clones when cells were transfected with the AGBL5-eGFP expression construct further suggest that the ciliary changes result from loss or reduction of functional AGBL5. AGBL5 and/or hyperglutamylation may therefore be targets for therapy in patients with defects in this gene, or other forms of retinal degeneration that involve changes in basal body or ciliary glutamylation levels, such as those with mutations in *TTLL5*, a tubulin glutamylase in which mutations cause cone-rod dystrophy.

### Investigation of druggable targets to rescue cilia defects in AGBL5^-/-^ cell lines

**Fig. 5:**
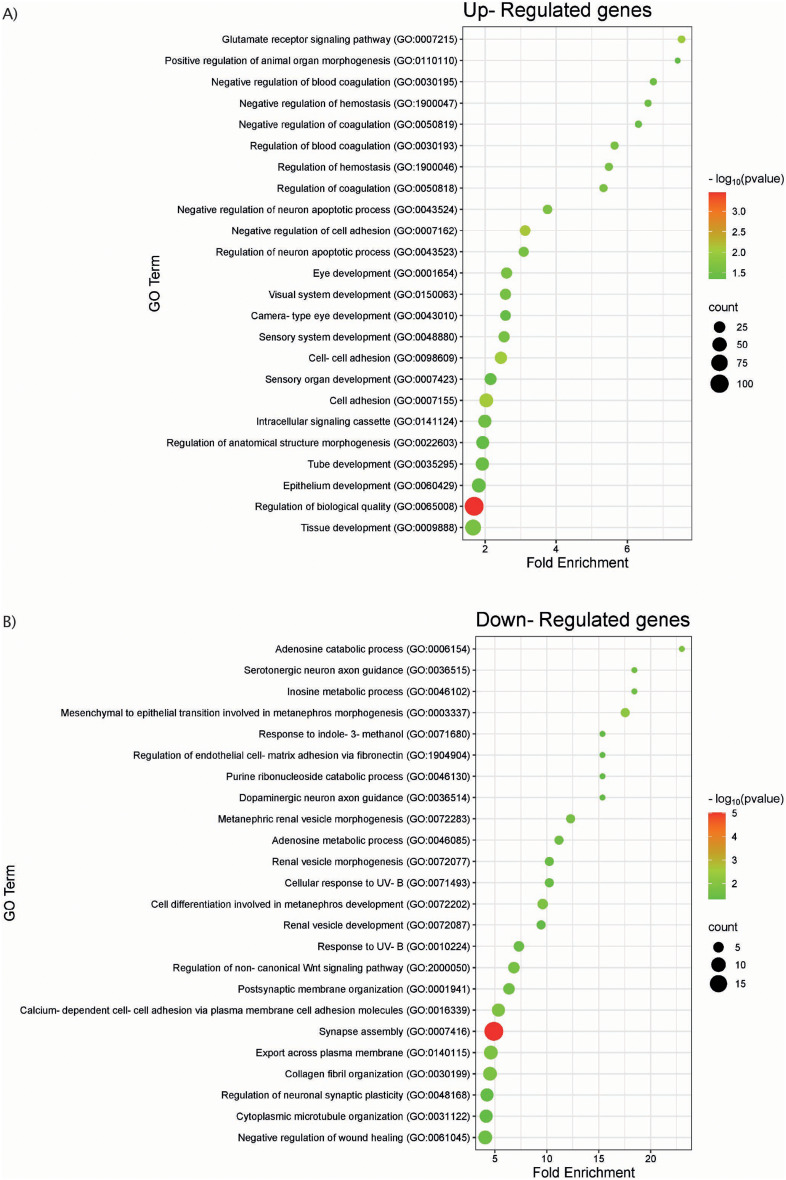
Gene Ontology enrichment analysis of differentially expressed genes in in *AGBL5*^*−/−*^ clones. The top 25 GO terms are shown on the Y axis. Bubble size represents the number of genes observed in the dataset, and the colour key shows intensity of colour relative to the -log(FDR-adjusted p-values) for each GO term. **a**) GO enrichment analysis of genes upregulated in *AGBL5*^*−/−*^ cells. **b**) GO enrichment analysis of genes downregulated in *AGBL5*^*−/−*^ cells

As the rescue of ciliogenesis defects and hyperglutamylation were so marked in the cells which were transfected with AGBL5-eGFP, we were interested to explore druggable targets for rescue of the phenotype through other pathways. We carried out analysis of RNAseq data from replicates of the ARPE19 WT and *AGBL5*^−/−^ clones to identify differentially expressed genes and pathways in *AGBL5*^−/−^ clones. Differential gene expression analysis of RNA-Seq data showed a total of 1,217 differentially expressed genes, of which 491 were upregulated and 726 genes were downregulated. Gene Ontology (GO) enrichment analysis of both groups of genes using the annotation data set for GO biological process, showed enrichment of the terms shown in Fig. 5a and b for upregulated and downregulated genes in *AGBL5*^−/−^ cells respectively. Notably, there is enrichment of genes involved in Wnt signalling, glutamate receptor signalling, nervous system development, synapse assembly and regulation, neuron apoptosis, cell adhesion, and eye/visual system development.

**Fig. 6:**
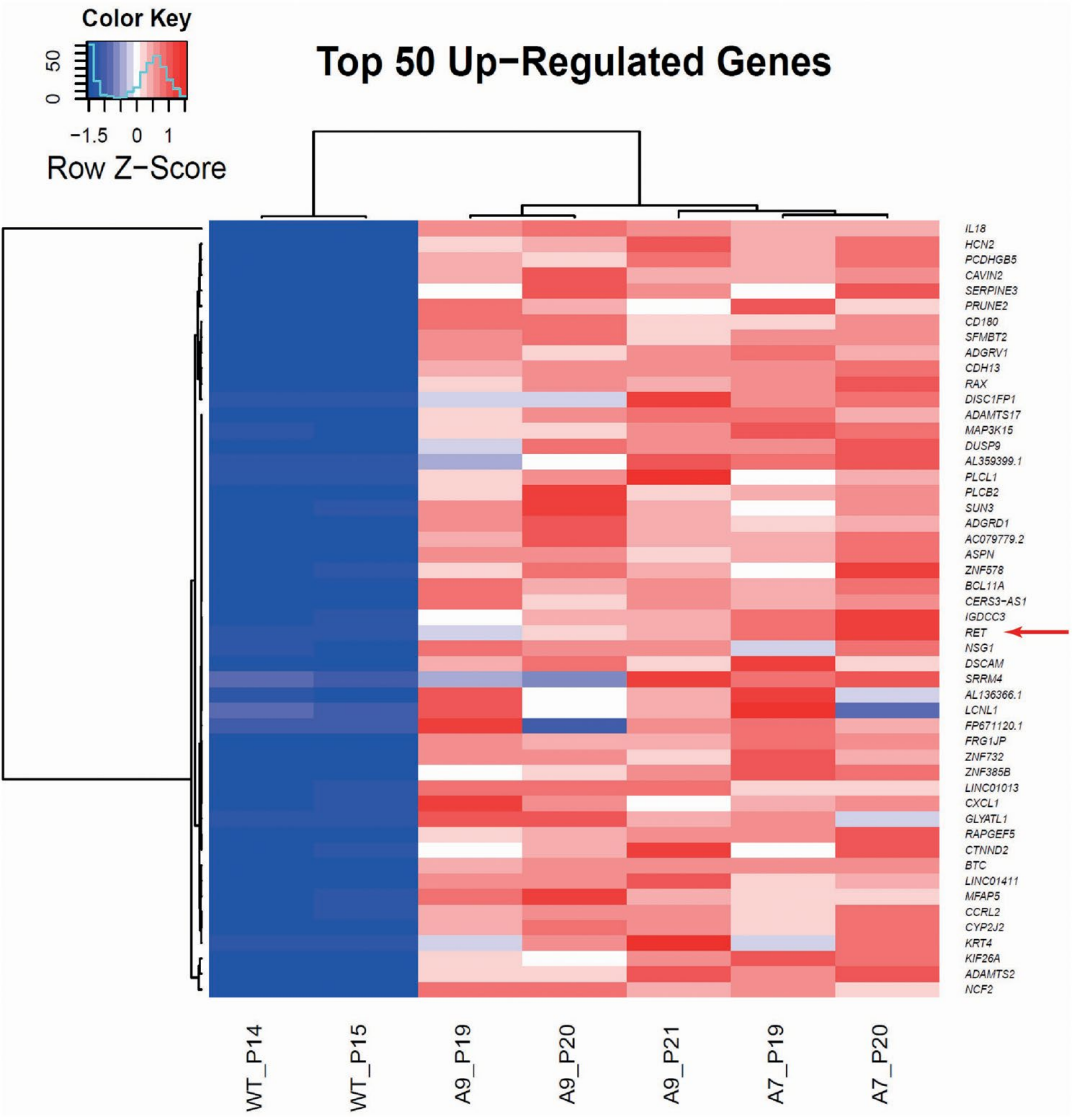
Identification of druggable targets for rescue of *AGBL5*^*−/−*^ clones. Heatmap showing the top 50 up-regulated genes in *AGBL5*^*−/−*^ clones A7 and A9 compared to ARPE19 WT cells. Colour key shows intensity of colour relative to the z score for each row. Arrow points at *RET*, a potential druggable target for rescue of ciliogenesis and loss and hyperglutamylation in *AGBL5*^*−/−*^ mutant line

Genes upregulated in *AGBL5*^−/−^ cells were sorted in descendant order based on the log fold change (logFC) (Fig. 6a). The top 50 genes were further studied for pharmacological inhibitors using the canSAR and GeneCards^®^ database (https://www.genecards.org/, accessed in April 2022). The search resulted in drugs associated to 10 genes, approved for used in humans. However, only 1 of these genes, *RET* (position 25th, logFC 3.72), was found to have associated drugs that inhibit the target gene/protein with high specificity, being Selpercatinib (LOXO-292) the most highly selective and potent approved small molecule that inhibits RET (124). Of interest, some of the GO terms associated with RET include neurone maturation (GO:0042551) and retina development in camera-type eye (GO:0060041).

Initial experiments focused on using the small molecule, Selpercatinib, to inhibit activation (phosphorylation) of RET. The effect of this drug was studied by cell proliferation assay MTS, trypan blue and western blot. However, effects in cells viability and proliferation were observed without changes in phosphorylation of the protein of interest (data not shown). Instead, we carried out CRISPR/Cas knockout of *RET* in *AGBL5* WT and *AGBL5*^−/−^ clones. *RET*^−/−^ was confirmed by Sanger sequencing (Supplementary Figure S3a) and evaluated by western blot and immunocytochemistry, but western blot showed no reduction in RET protein expression in the polyclonal lines (Supplementary Figure S3b) and no changes were detected in cilia by immunocytochemistry (Supplementary Figure S3c, d, e).

Although we could question the specificity of the RET antibody, two different antibodies were used, a monoclonal antibody for RET (C31B4), and polyclonal for Phospho-RET (Tyr905), which detected the same band and of the expected molecular weight. Therefore, it is possible that *RET*^*−/−*^ cells compensate for the indels introduced by using an alternative splice site which retains the main reading frame, as occurred with the *AGBL5*^*−/−*^ clones.

As an alternative approach to rescuing ciliogenesis and hyperglutamylation defects in *AGBL5*^−/−^ clones we targeted an initiating glutamylase enzyme responsible for the initiation of glutamate side chains, hypothesizing that we may prevent the formation of chains that cannot be removed in the absence of AGBL5 activity. Among the four tubulin-initiating glutamylases, *TTLL5* was the most highly expressed in ARPE19 cells, detected by RNAseq analysis (Supplementary Table S5), and therefore we selected this as a potential target to restore normal glutamylation levels in *AGBL5*^*−/−*^ cells. *TTLL5* localises to the basal bodies of photoreceptors [34], glutamylates RPGR [35], α-tubulin and β-tubulin after saturation of glutamylation sites in α-tubulin [36]. Although mutations in *TTLL5* cause cone-rod dystrophy in humans [34] and reduced male fertility [37] we hypothesized that on an AGBL5 knockout background, reduction in TTLL5 levels could be potentially therapeutic.

**Fig. 7:**
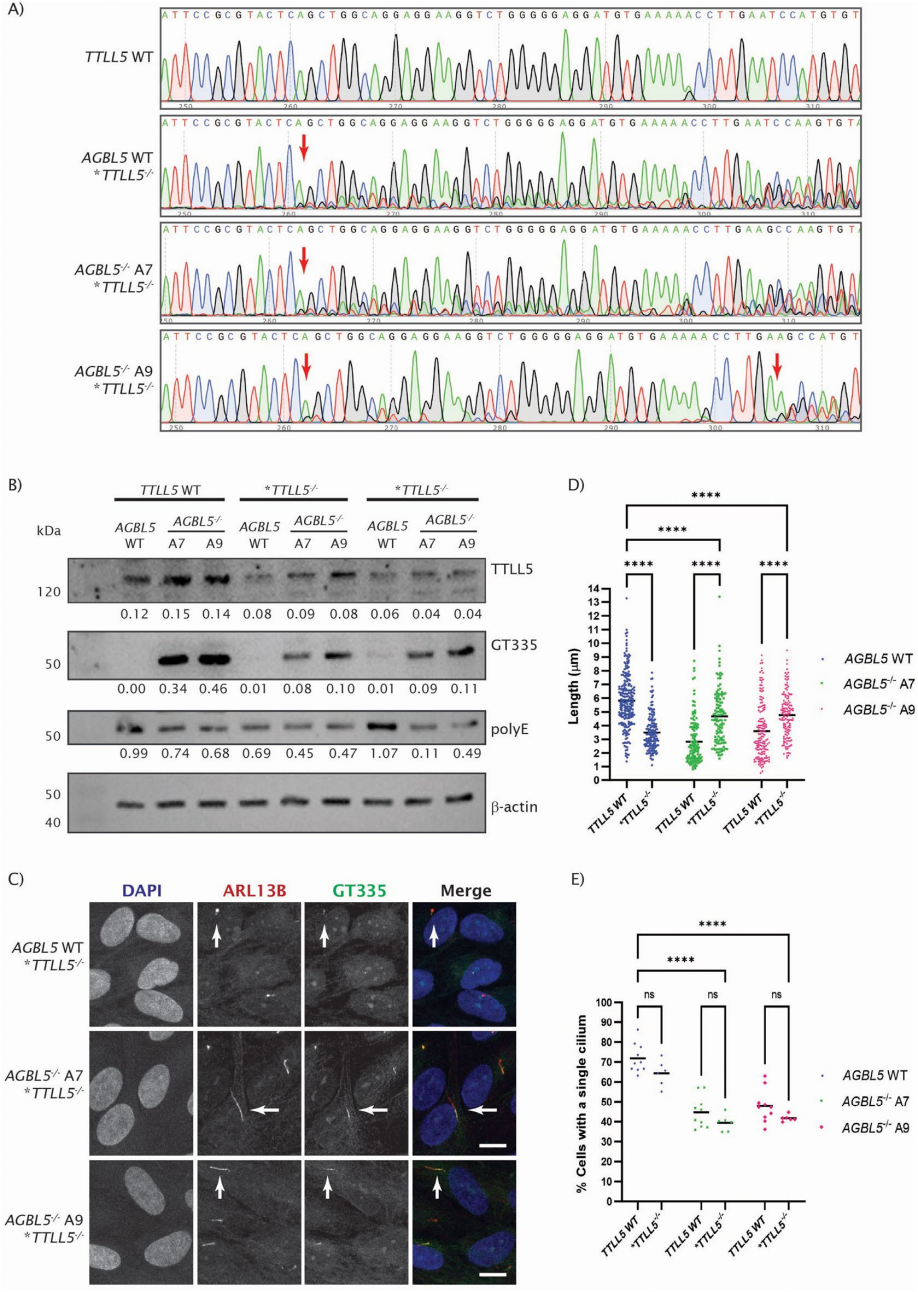
Rescue of ciliogenesis and hyperglutamylation in *AGBL5*^*−/−*^ mutant line through CRISPR/Cas9 knockdown of initiating glutamylase, TTLL5. **a**) Sanger sequencing traces of WT *TTLL5* the *TTLL5*^*−/−*^ pools. Arrows point at the sites where mutated clones were modified. **b**) Western blot image showing expression of TTLL5, glutamylated tubulin (GT335), polyglutamylation (PolyE) and β-actin loading control in *AGBL5* WT and *AGBL5*^*−/−*^ clones with WT *TTLL5*, and two biological replicates of *TTLL5*^*−/−*^ pools. Densitometry values were normalised to the loading control. **c**) Immunocytochemistry images showing rescue of cilia defects in *AGBL5*^*−/−*^/*TTLL5*^*−/−*^ pools. Arrows point to cilia. Scale bar 10 μm. **d**) Dot plot of individual measures of cilia length in WT *TTLL5* and *TTLL5*^−/−^ pools. Bars represent the mean, **** *p* < 0.0001. **e**) Dot plot quantifying the cilia number in WT *RET* and *RET*^−/−^ pools. Each dot represents a field of view. Bars represent the mean, **** *p* < 0.0001, ns not significant

We generated polyclonal *TTLL5* CRISPR knockout lines of in WT and *AGBL5*^−/−^ clones. The mutation in the KO polyclonal populations were confirmed by Sanger sequencing (Fig. 7a) and Inference of CRISPR Edits (ICE) analysis (Synthego) and the effect of the genomic mutation was evaluated by western blot (Fig. 7b) and immunocytochemistry (Fig. 7c, d, e). Western blot results showed reduction of TTLL5 protein rather than loss of TTLL5 protein corresponding to the fact that we generated a polyclonal pool of cells of which some had a TTLL5 indel and others did not. Reduction of TTLL5 levels in this cell population was accompanied with significant reduction of tubulin glutamylation as was observed using GT335, and a tendency to decrease in polyE signals, although the latter was variable across replicates (Fig. 7b). Immunocytochemistry showed reduction of ciliogenesis in *AGBL5* WT/*TTLL5* KO population, partial rescue of cilia length in *AGBL5*^−/−^/*TTLL5*^−/−^ cells (Fig. 7c, d)., but no significant changes in ciliogenesis (Fig. 7e).

**Fig. 8:**
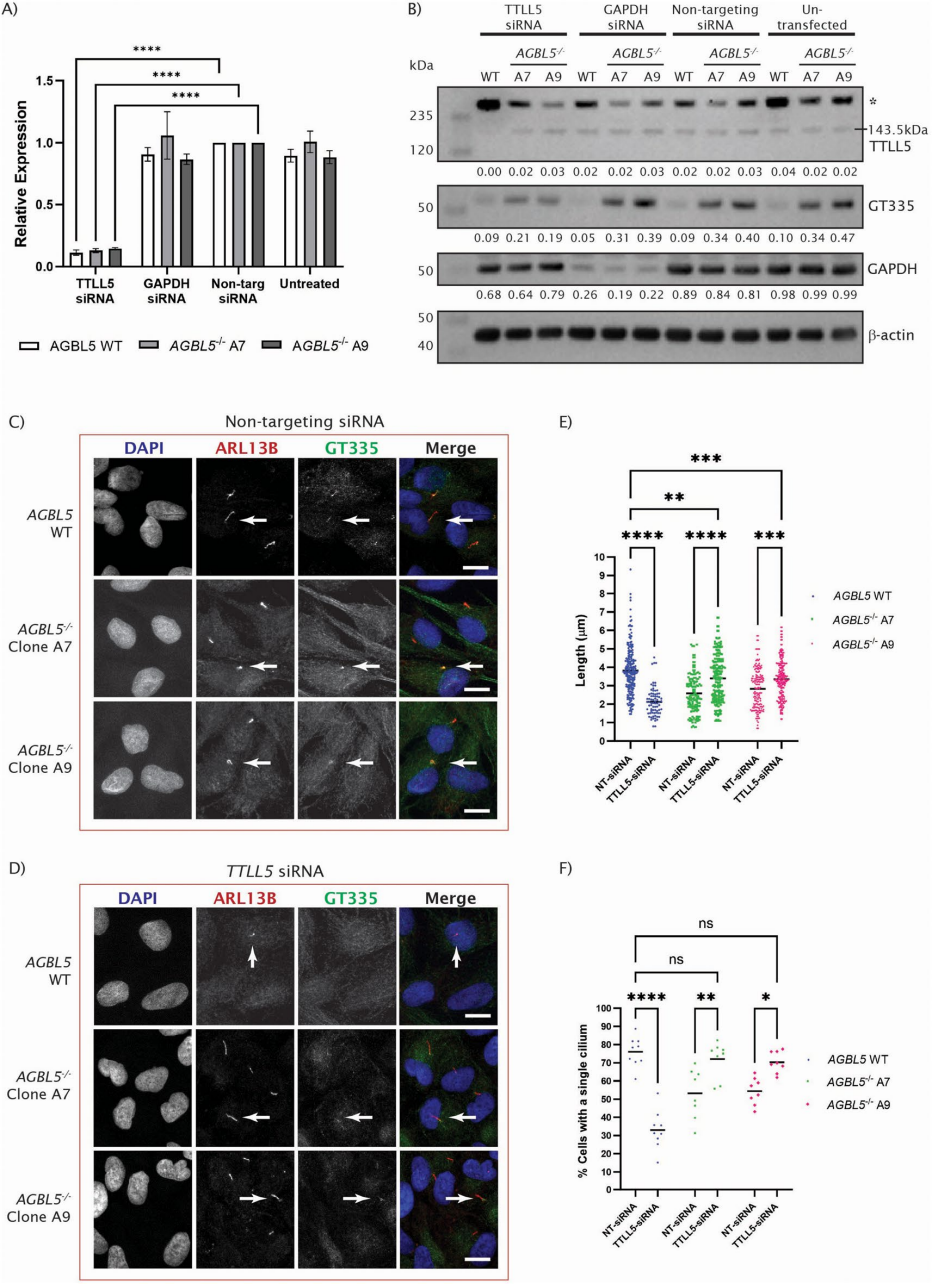
Rescue of ciliogenesis and hyperglutamylation in *AGBL5*^*−/−*^ mutant lines through siRNA knockdown of initiating glutamylase, *TTLL5. ***a**) qPCR results showing relative expression of *TTLL5* (normalised to *ACTB*) after 48 h of treatment with siRNA against *TTLL5*, *GAPDH*, non-targeting controls and untreated cells. Results are presented as the mean of three biological replicates, and the standard error of the mean; **** *p* < 0.0001. **b**) Western blot image showing expression of TTLL5 (line indicates position of 143 kDa TTLL5 band; asterisk denotes higher band of unknown origin), glutamylated tubulin (GT335), GAPDH and β-actin loading control in *AGBL5* WT and *AGBL5*^*−/−*^ clones treated with *TTLL5*^*−/−*^ siRNA, GAPDH siRNA, non-targeting control siRNA and untreated cells. Densitometry values were normalised to the loading control and showed no change in TTLL5 levels but confirms reduction of GT335 signals after 120 h. **c**) Immunocytochemistry images showing no changes in the cilia phenotype in *AGBL5* WT and *AGBL5*^*−/−*^ mutant lines treated with non-targeting siRNA for 120 h. Arrows point to cilia. Scale bar 10 μm. **d**) Immunocytochemistry images showing rescue of the cilia defects in *AGBL5*^*−/−*^ mutant lines treated with *TTLL5* siRNA for 120 h. Arrows point to cilia. Scale bar 10 μm. **e**) Dot plot of individual measures of cilia length in cells treated with non-targeting (NT) siRNA and *TTLL5* siRNA for 120 h. Bars represent the mean, **** *p* < 0.0001, *** *p* < 0.001, **p < 0.01. **f**) Dot plot quantifying the cilia number in cells treated with non-targeting (NT) siRNA and *TTLL5* siRNA for 120 h. Each dot represents a field of view. Bars represent the mean, **** *p* < 0.0001, **p < 0.01, *p = 0.026, ns not significant

Due to the advances in use of antisense oligonucleotides (ASOs) in treatment of rare disease, including rare retinal diseases [38], we were interested to test whether an siRNA approach to reducing *TTLL5* levels could rescue the ciliogenesis and glutamylation defects observed in *AGBL5*^−/−^ clones. Gene silencing using siRNA was initially performed and assay by western blots up to 72 h after transfection, without observing any effect on TTLL5 or GT335 expression, whilst GAPDH siRNA positive control experiments confirmed the knockdown experiments were working, and knockdown was observed at RNA level, (Fig. 8a). Extending the siRNA knockdown treatment to 120 h enabled us to observe changes at protein level (Fig. 8b). 120 h after *TTLL5* siRNA transfection we still did not observe reduction in TTLL5 expression by western blot, but we detected a decrease in tubulin glutamylation (GT335) (Fig. 8b). Immunocytochemistry showed cilia deficiency in *AGBL5* WT/*TTLL5*^−/−^ populations, and rescue of cilia length in *AGBL5*^−/−^/*TTLL5*^−/−^ cells (Fig. 8c, d, e), consistent with the results obtained by CRISPR KO of *TTLL5*, and it also revealed rescue of ciliogenesis after *TTLL5* siRNA treatment (Fig. 8f) which was not observed in the CRISPR experiment.

### AGLB5 protein interactants

As of December 2023, 25 protein interactions for AGBL5 had been identified in different studies (Supplementary Table S6) [39–45]. However, most of these interactants were detected as part of large-scale or proteomic studies unrelated to the retina. In order to obtain preliminary data of potential AGBL5 protein interactants and mechanisms by which AGBL5 regulates ciliogenesis and cilia length in the RPE, and considering the lack of AGBL5 antibodies suitable and/or validated for immunoprecipitation, we used an eGFP nanobody to pull down transiently expressed exogenous AGBL5-eGFP in ARPE19 WT cells and used mass spectrometry to identify proteins precipitated. Protein from cells transfected with pmaxGFP were used as a transfection control.

**Fig. 9:**
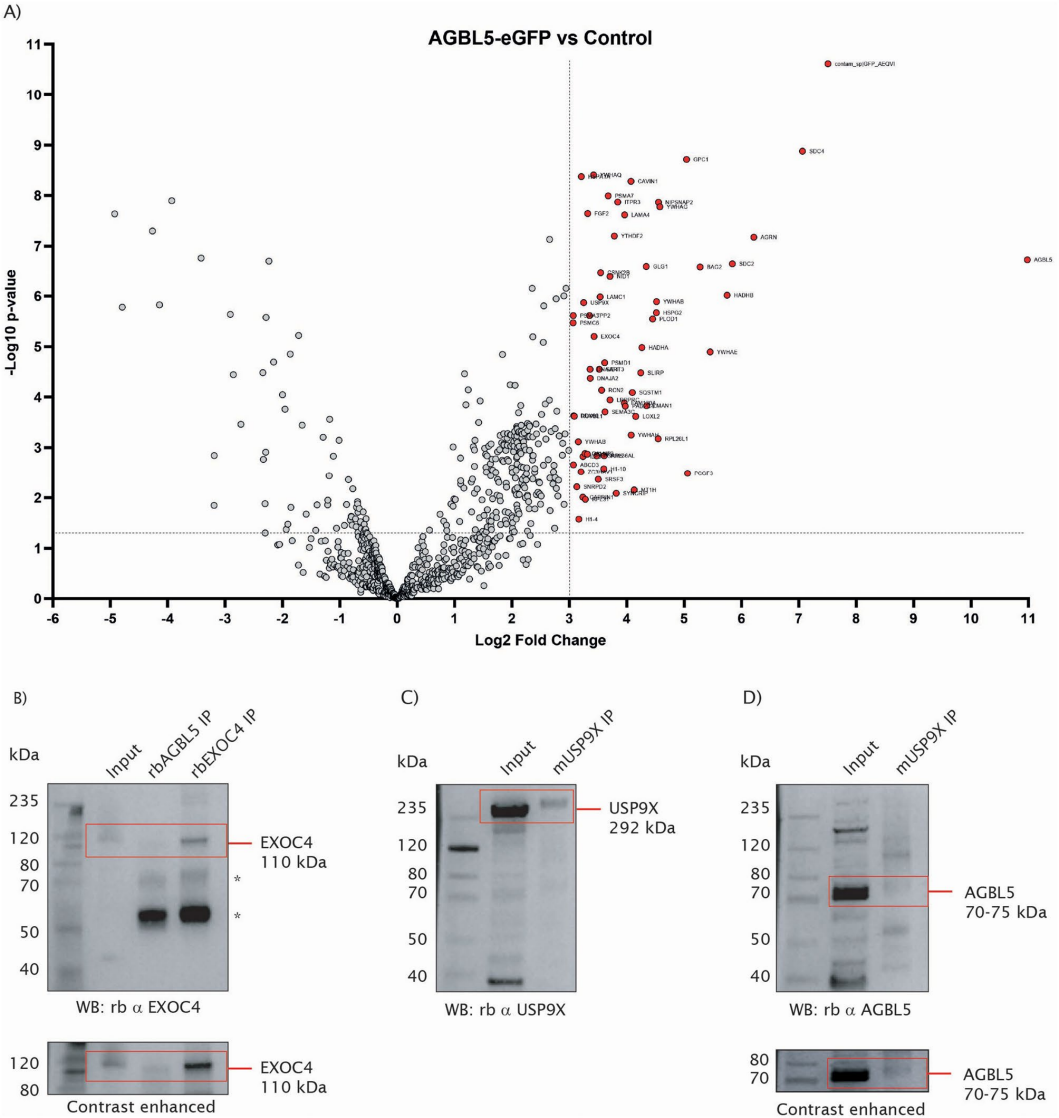
AGBL5-eGFP protein interactants identified by co-IP and MS. **a**) Volcano plot showing proteins identified from 6 replicates of each sample. Proteins with negative log10 p value > 1.3 (p value < 0.05 in linear scale) and log2FC values > 3 are shown in red. **b**) Co-immunoprecipitation of endogenous AGBL5 and EXOC4, using a rabbit AGBL5 antibody, and a rabbit EXOC4 antibody. * signals predicted to originate from the antibody. **c**) Co-immunoprecipitation of USP9X using a mouse USP9X antibody, for detection of USP9X. **d**) Co-immunoprecipitation of USP9X using a mouse USP9X antibody, for detection of AGBL5

A total of 925 proteins were obtained by mass spectrometry analysis of the co-IP products, including common contaminants (Supplementary Table S7). Maximal Label-free Quantification (MaxLFQ) intensity values were used to perform a comparative analysis of the proteins identified in ABGL5-eGFP samples versus the controls, and the fold change (FC) in log2 scale was calculated based on the AGBL5-eGFP/control ratio. As we were interested in proteins highly enriched in the AGBL5-eGFP samples, we focused on proteins with log2 FC > 3. The 64 proteins that passed this threshold are represented with red bubbles in Fig. 9a. Two proteins in our data were identified in previous studies: the Polycomb Group Ring Finger 3 PCGF3 and the chaperone DNAJA2. The data was also screened for presence of cilia proteins; we found YWHAE, YWHAG, YWHAQ, SLIRP, SQSTM1 and CSNK2B, which were reported in the CiliaCarta [46], EXOC4 and RUVBL1 reported in the CiliaCarta and the SCGSv2 list [47], and USP9X in the SCGSv2 only. For validation of interactions, the cilia proteins exocyst complex component 4 EXOC4 and ubiquitin specific peptidase 9 X-linked USP9X were selected.

We performed co-IP of endogenous AGBL5 and EXOC4 and western blotting to validate their interaction (Fig. 9b). We were able to see a clear 110 kDa band in the input fraction and in the EXOC4 co-IP product, confirming that EXOC4 was pulled down successfully. In the AGBL5 co-IP product, a faint band of approximately the same size was also observed, and contrast adjustment of the image allowed better visualisation. This result suggests that EXOC4 precipitated together with the endogenous AGBL5. Additional bands were observed in both lanes containing IP products (marked with *), which may be signals from the secondary antibody binding to rabbit heavy chain immunoglobulin, or non-specific binding of the EXOC4 antibody.

To test USP9X interaction, we performed co-IP of USP9X and observed the expected 292 kDa band in both the input fraction and in the IP product (Fig. 9c), which confirms successful immunoprecipitation of USP9X. Incubation with the AGBL5 antibody also showed the expected ~ 70–75 kDa band in the input fraction, and a faint blurry band in the USP9X co-IP product (Fig. 9d), suggesting that AGBL5 may have been coimmunoprecipitated with USP9X.

## Discussion

Association of *AGBL5* with RP in humans was first reported in 2015 and since then evidence for its role in retinal disease has increased and it is now part of the green list of the Retinal Disorders panel (Version 6.0) in Genomics England PanelApp (https://panelapp.genomicsengland.co.uk/panels/307/ accessed in August 2024), and is used in genetic screening for retinal disorders in the UK National Health Service NHS. There remain no treatments, and the role of AGBL5 in the retina remains poorly understood. The aim of this study was to elucidate molecular mechanisms of disease and explore avenues for potential therapies in a convenient and easy way to manipulate human adult retinal epithelial cell line.

### AGBL5^-/-^ leads to excess of glutamate side chains without increase of the length

We generated two clonal *AGBL5*^*−/−*^ adult retinal epithelial cell lines, confirmed introduction of indels at the genomic locus, and knockout of WT RNA and protein in these cells. Consistent with the literature, we observed tubulin hyperglutamylation when AGBL5 was depleted [9, 13, 15–17], whilst in the WT cells it was low or undetectable. These cells recapitulate the cellular phenotype observed in *Ccp5*^*−/−*^ mice.

Hyperglutamylation may be due to an increase in the number of glutamylated sites, the length of the glutamate side chains, or both. GT335 antibody detects specifically the branching points of the glutamate side chain, independent of the length, which means that it can detect tubulin mono- and polyglutamylation. We hypothesised that the hyperglutamylation associated with loss of AGBL5 would primarily involve monoglutamylation, as other published studies have shown that in *Ccp5*^*−/−*^ knockout mouse mutant retinas there is evidence of hyperglutamylation with no change in levels of polyglutamylation, identified using poly(E) antibody which detects glutamate chains of 4 or more residues [16]. However, blotting of lysates from our cell lines with this poly(E) polyglutamylation antibody unexpectedly showed a decrease in polyglutamylation. This suggests that the number of glutamylated sites increases due to AGBL5 depletion (which removes branching point glutamates preferentially) as observed with the GT335 antibody, but does not increase polyglutamylation signals, suggesting that other mechanisms prevent further elongation of the glutamate side chains. We hypothesise that the difference in our findings compared to the findings in whole mouse retinal lysates may be down to either species-specific differences, or cell type specific differences, as we are investigating immortalised adult human retinal pigmented epithelial cell lines as opposed to whole retinal lysates from mice. We further hypothesise that other CCPs expressed in ARPE19 cells, such as AGTPBP1, which is responsible for removal of side chain glutamates, may be regulating the length of the glutamate side chains rather than AGBL5. RNA-seq data from our ARPE19 WT cells showed that *AGTPBP1*, which encodes a side chain deglutamylase [48, 49] is the second most abundant CCP expressed in ARPE19 cells, whilst *AGBL1* (also known as *CCP4*) was not expressed. Experiments to deplete AGTPBP1 could confirm if this protein is the mechanism by which the cells are still regulating glutamate side chains length.

### ARPE19 AGBL5^-/-^ cells display a cilia-deficient phenotype

Either the protein loss, its reduction, or partial functionality, are associated with phenotypic changes in our *AGBL5*^−/−^ clones compared to the WT cells. Consistent with a recent report showing shortening of the photoreceptor cilium in *Ccp5*^*−/−*^ mice [16], we found that disruption of *AGBL5* is consistently associated with shorter cilia and reduced ciliogenesis, as the percentage of ciliated cells was lower in the *AGBL5*^−/−^ clones (Fig. 3d). This effect was observed in all the immunofluorescence experiments performed, including the GFP transfection assays (Fig. 4), *RET* CRISPR KO (Fig. 6), and control siRNA knockdown experiments (Fig. 7). It is important to acknowledge that the ARPE19 cells used in this study are a simple, monolayer culture model which do not phenotypically or transcriptionally fully recapitulate in vivo retinal pigment epithelium, or retinal cells which are more relevant to the disease pathogenesis in humans. However, they are a valuable, easy to manipulate human cell model which can conveniently be used to study the molecular function of AGBL5. These cells could also easily be exploited in large-scale high-throughput functional genomics screens or drug screens to accelerate discovery of drugs for treating AGBL5-associated RP, which would be difficult to achieve using human stem cell-derived organoids or mouse models. To explore this further, we carried out some targeted experiments to attempt to rescue cilia-loss and hyperglutamylation phenotypes observed in the *AGBL5*^*−/−*^ cells.

### Gene augmentation or depletion of TTLL5 can rescue hyperglutamylation and cilia deficiency

We tested the potential of gene augmentation therapy in ARPE19 *AGBL5*^*−/−*^ cells by transient expression of exogenous AGBL5. Transfection with an AGBL5-eGFP expression construct was able to produce robust rescue of hyperglutamylation, cilia length and ciliogenesis rates in *AGBL5*^*−/−*^ cells. In addition, overexpression of AGBL5 in *AGBL5* WT cells did not negatively affect the cilia phenotype. Besides reducing total glutamylation in *AGBL5*^*−/−*^ cells (assessed using GT335 antibody), the expression of exogenous AGBL5 also reduced polyglutamylation significantly (4 or more glutamates, detected using the polyE antibody) in *AGBL5* WT and *AGBL5*^*−/−*^ cells. However, the presence of long cilia in *AGBL5* WT cells after transfection and the high percentage of cells producing cilia indicates that long tubulin glutamate side chains may not be as critical in ciliogenesis as the number of side chains, at least during the first stages of cilia formation and elongation.

Some studies have suggested that AGBL5 only removes γ-linked glutamates (branching point), which are accessible only after α-linked (side chain) glutamates have been removed [14, 48]. However, another study demonstrated that AGBL5 can remove α- and γ-linked glutamates, but the rate of α-linked cleavage is significantly slower [7]. Our transfection results support a dual deglutamylase function of AGBL5, as overexpression of AGBL5 resulted in reduction of both the number of glutamate side chains and the length of these chains, potentially increasing the speed of the removal of α-linked glutamates to shorten the side chains and facilitating subsequent removal of the branching point glutamate.

Glutamylation plays an important role in the regulation of IFT, which is required for trafficking of ciliary precursors and for the assembly of growing microtubules [50–52]. Appropriate levels of glutamylation facilitate kinesin-2-mediated anterograde IFT, which could be inhibited in the hyperglutamylation state. Mislocalisation of cilia proteins such as intraflagellar transport proteins is observed in Ccp5-/ mice [17]. It would be interesting to study further whether IFT is disturbed in *AGBL5*^*−/−*^ ARPE19 cells. Tubulin hyperglutamylation also has been reported to lead to microtubule disassembly due to the binding of Spastin, a severing enzyme [53]. This is a mechanism which could be further explored in *AGBL5*^*−/−*^ ARPE19 cells. In addition to axoneme stability, glutamylation is essential for centriole stability [54], with reports of increased number of basal bodies in spermatids in mice lacking AGBL5/CCP5 [15], and is also critical for maintaining Sonic Hedgehog (Shh) signalling [6]. Of note, Wnt is known to antagonise Shh signalling [55], and our DGE analysis showed genes involved in Wnt signalling being differentially expressed. Therefore, *AGBL5* and/or hyperglutamylation could be explored as targets for therapy in patients with defects in this gene, or other forms of retinal degeneration that involve changes in basal body or ciliary glutamylation levels.

As well as CCPs such as AGBL5, TTLL proteins are involved in the regulation of tubulin glutamylation; TTLL4, 5, and 7 are responsible for initiation of glutamate side chains, and TTLL1, 6, 9, 11 and 13 are responsible for elongation [8, 56–58]. Therefore, reducing initiating glutamylases such as TTLL5, which has also been associated with hyperglutamylation and retinal degeneration [34], could restore the balance of glutamylation in *AGBL5* deficient cells. Indeed, our CRISPR and siRNA knockdown experiments showed that disruption of *TTLL5* negatively impacts ciliogenesis in ARPE19 *AGBL5* WT cells and leads to shorter cilia, whilst in *AGBL5*^*−/−*^ cells it rescues the cilia deficiency and reduces hyperglutamylation.

Taken together, these results suggest that delivery of a WT version of *AGBL5* or reducing expression of *TTLL5* could be a potential therapy for patients with RP associated with mutations in *AGBL5*. Delivery using AAV has been widely studied, but the main limitation of AAV lies in their capacity to carry approximately 5 kb of genetic material [59]. Nonetheless, *AGBL5* ORFs are short enough (2,658 bp for the long transcript and 2,154 bp for the short) to allow the construction of the vector within the size. On the other hand, knockdown of *TTLL5* could be further explored as a therapeutic option, for instance, using ASOs.

### EXOC4 and USP9X as putative interactants of AGBL5

Although this study supports hyperglutamylation as the main factor determining the cilia deficiency in *AGBL5*^*−/−*^ cells, important protein interactions for ciliogenesis regulation may be disrupted by different genetic variants, therefore, unveiling protein interaction and understanding how pathogenic mutations disrupts those interactions could help explain the pathogenesis of the disease. In this study, we identified EXOC4 and USP9X as potential interactants of AGBL5, although further experiments are required to validate these interactions confidently.

EXOC4 and EXOC5 (filtered out after applying the log2 FC > 3 cut off) have been described to play a role in development of the retina and ciliogenesis in photoreceptors [60]. USP9X, plays a role in ciliogenesis of primary cilia, and mediates the regulation of tight junctions’ formation in the polarization of epithelial cell during differentiation [61], an important process in the development of the RPE.

If validated, these interactions could provide significant insights into the mechanisms by which AGBL5 regulates ciliogenesis in the retina. The exocyst has been reported to be involved in ciliary transport and targeting of transmembrane proteins to the cilium [62, 63], as well as soluble proteins [64]. There exists the possibility that an interaction between AGBL5 and exocyst proteins is required for its localisation to the cilium. The other potential interactant, USP9X, has been found to regulate deubiquitination cycles of ciliogenesis regulators [65]. It is then possible that AGBL5 is a substrate of USP9X and its deubiquitination regulates its localisation to the cilium, protein interactions, or degradation cycles to maintain glutamylation levels at different stages of cilia assembly, resorption or maintenance.

Furthermore, it has been demonstrated that non-tubulin proteins are also targets of glutamylation and deglutamylation [10, 66, 67]. AGBL5 deglutamylation activity in particular, is required to activate cyclic GMP-AMP synthase and subsequent interferon response during viral infection [10]. Therefore, the possibility that EXOC4 and/or USP9X are also subjected to glutamylation should not be excluded, as they may require deglutamylases such as AGBL5 for correct function.

### Summary

In summary, we present two clonal *AGBL5*^*−/−*^ ARPE19 cell lines which recapitulate features of hyperglutamylation and ciliary axonome shortening recently described in Ccp5-/- mice. We use this convenient human cell knockout model to investigate the protein interactants of AGBL5 and demonstrate the potential for rescue of cellular phenotypes using AGBL5 overexpression or TTLL5 knockdown. These cells represent an opportunity for further characterization of the molecular mechanism of disease associated with AGBL5 loss and a convenient and powerful model system suitable for exploitation in high-throughput phenotypic screens such as high content imaging screens, to explore the functional effect of AGBL5 patient mutations for characterisation of variants of unknown clinical significance, or for testing of potential therapies.

## Supplementary Information

Below is the link to the electronic supplementary material.

Supplementary Figure S1. *AGBL5*^*−/−*^ mutant cell line protein expression characterisation. Densitometry analysis of AGBL5 (A), GT335 (B), and PolyE (C) signals detected by western blot in *AGBL5* WT and *AGBL5*^*−/−*^ clones in 2 biological replicates with 4 different conditions, normalised against the β-actin loading control. Results were plotted as individual values with the median and interquartile range, and statistical analysis was performed using the nonparametric Mann Whitney test. **** *p* < 0.001, **p < 0.01

Supplementary Figure S2. Aberrant splicing in ARPE19 *AGBL5*^*−/−*^ clones. a) Sanger sequencing traces of *AGBL5* WT showing correct exon3-exon4 junction splicing. b-e) Sanger sequencing traces of *AGBL5*^*−/−*^ clones showing aberrant splicing, including partial skipping of exon 4 (B), total skipping of exons 3 to 7 and partial skipping of exons 2 and 8 (C), total skipping of exons 3 to 8 and partial skipping of exons 2 and 9 (D), and partial skipping of exon 3 with inclusion of an intronic region (E). Note that the exon 4 sequence showed in panel is the same detected and showed in Fig. 1B

Supplementary Figure S3. Testing a druggable target for rescue of ciliogenesis and loss and hyperglutamylation in *AGBL5*^*−/−*^ mutant line through differential gene expression analysis. (a) Druggable targets RET: Sanger sequencing traces of WT *RET* the *RET*^*−/−*^ pools. Arrows point at the sites where mutated clones were modified. (b) Druggable targets RET: western blot image showing expression of RET, AGBL5 and β-actin loading control in *AGBL5* WT and *AGBL5*^*−/−*^ clones with WT *RET*, and two biological replicates of *RET*^*−/−*^ pools. Densitometry values were normalised to the loading control. (c) Immunocytochemistry images showing lack of rescue of cilia defects in *RET*^*−/−*^ pools. Arrows point to cilia. Scale bar 10μm. (d) Dot plot quantifying cilia length in WT *RET* and *RET*^−/−^ pools. Bars represent the mean, **** *p* < 0.0001, ns not significant. (e) Dot plot quantifying the cilia number in WT *RET* and *RET*^−/−^ pools. Each dot represents a field of view. Bars represent the mean, ns not significant

Supplementary Tables S1–S6. Supplementary Table S1: Primer sequences used for PCR, RT-PCR, qPCR and sequencing. Supplementary Table S2: Primary antibodies used in western blot and fluorescent ICC. Supplementary Table S3: Secondary antibodies used in western blot and fluorescent ICC. Supplementary Table S4: AGBL5 transcript expression as transcripts per million reads (TPM) in WT and AGBL5-/- cells. Supplementary Table S5: TTLL gene expression as transcripts per million reads (TPM) in WT and AGBL5-/- A7 and A9 clones. Supplementary Table S6. AGBL5 protein interactants reported in the literature and references

Supplementary Table S7. Mass spec results of proteins purified from 6 replicates of AGBL5-eGFP co-immunopreceiptation experiment in which an eGFP nanobody was used to pull down transiently expressed exogenous AGBL5-eGFP in ARPE19 WT cells, compared to 6 replicates of eGFP control co-immunopreceiptation experiment in which pmaxGFP was expressed and an eGFP nanobody was used to pull down proteins in ARPE19 WT cells

## Data Availability

Cell lines are available upon request. RNAseq FASTQ files are deposited in the European Nucleotide Archive and are accessible under accession numbers PRJEB96053 (Project) and ERP178802 (Study).
