# Supplementary figures and images for: Rescue of ciliogenesis and hyperglutamylation mutant phenotype in AGBL5−/− cell model of retinitis pigmentosa
Source: BMC Mol Cell Biol. 2025 Sep 9;26:27. doi: 10.1186/s12860-025-00551-x (PMC12418683; doi:10.1186/s12860-025-00551-x)

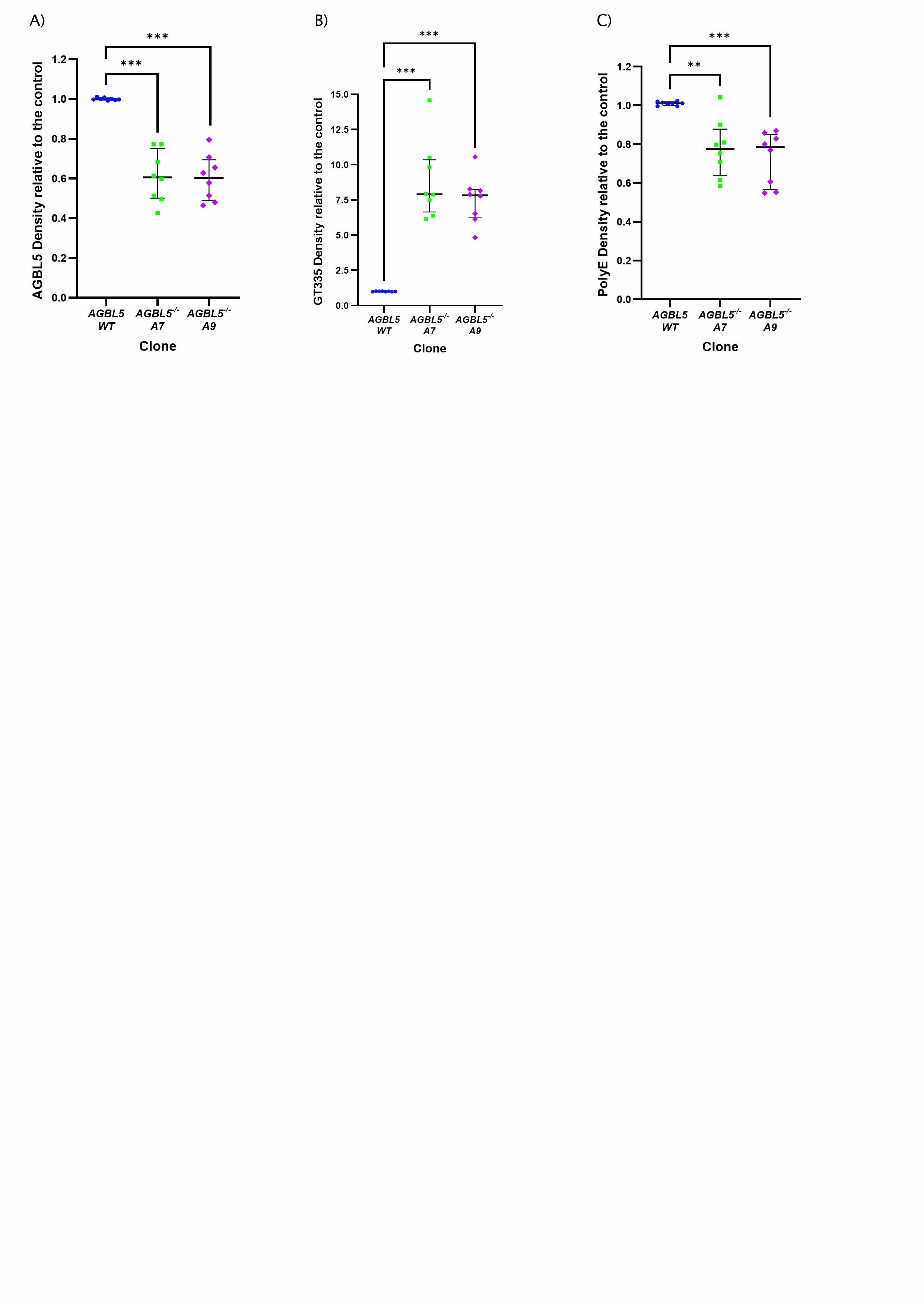

Supplement: Supplementary file 1 — Supplementary Figure S1. AGBL5−/− mutant cell line protein expression characterisation. Densitometry analysis of AGBL5 (A), GT335 (B), and PolyE (C) signals detected by western blot in AGBL5 WT and AGBL5−/− clones in 2 biological replicates with 4 different conditions, normalised against the β-actin loading control. Results were plotted as individual values with the median and interquartile range, and statistical analysis was performed using the nonparametric Mann Whitney test. **** p < 0.001, **p < 0.01 [file 12860_2025_551_MOESM1_ESM.jpg]

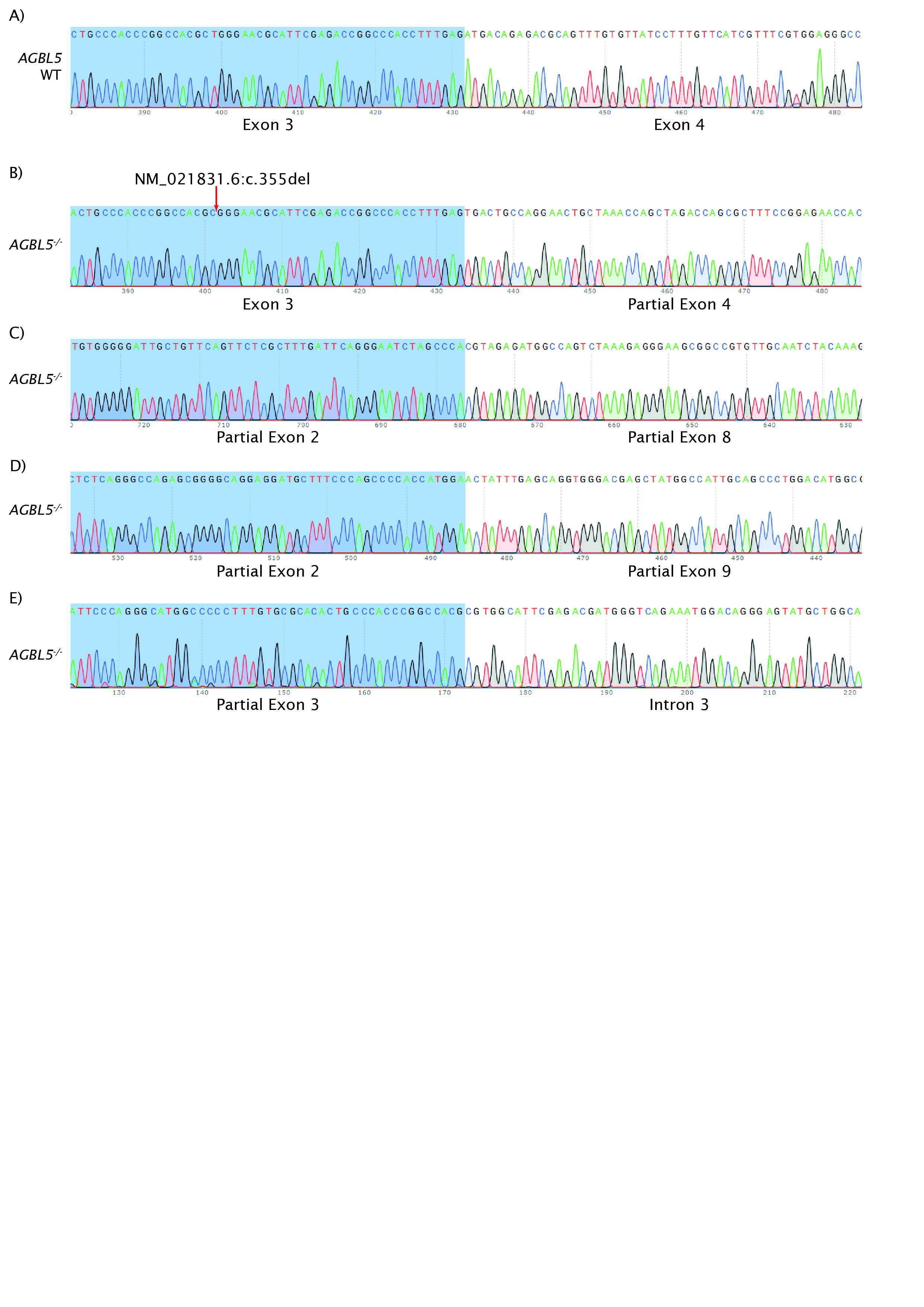

Supplement: Supplementary file 2 — Supplementary Figure S2. Aberrant splicing in ARPE19 AGBL5−/− clones. a) Sanger sequencing traces of AGBL5 WT showing correct exon3-exon4 junction splicing. b-e) Sanger sequencing traces of AGBL5−/− clones showing aberrant splicing, including partial skipping of exon 4 (B), total skipping of exons 3 to 7 and partial skipping of exons 2 and 8 (C), total skipping of exons 3 to 8 and partial skipping of exons 2 and 9 (D), and partial skipping of exon 3 with inclusion of an intronic region (E). Note that the exon 4 sequence showed in panel is the same detected and showed in Fig. 1B [file 12860_2025_551_MOESM2_ESM.jpg]

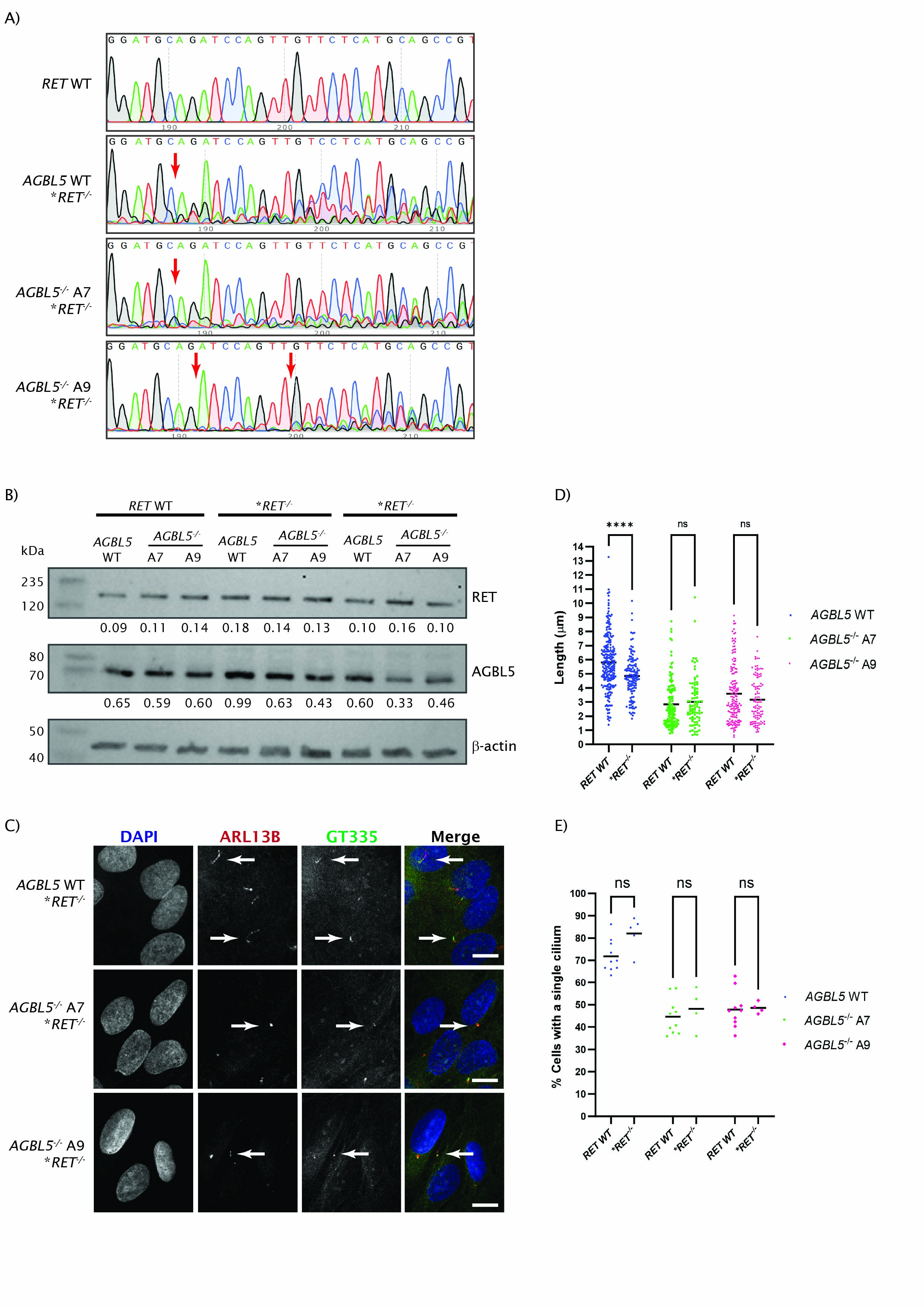

Supplement: Supplementary file 3 — Supplementary Figure S3. Testing a druggable target for rescue of ciliogenesis and loss and hyperglutamylation in AGBL5−/− mutant line through differential gene expression analysis. (a) Druggable targets RET: Sanger sequencing traces of WT RET the RET−/− pools. Arrows point at the sites where mutated clones were modified. (b) Druggable targets RET: western blot image showing expression of RET, AGBL5 and β-actin loading control in AGBL5 WT and AGBL5−/− clones with WT RET, and two biological replicates of RET−/− pools. Densitometry values were normalised to the loading control. (c) Immunocytochemistry images showing lack of rescue of cilia defects in RET−/− pools. Arrows point to cilia. Scale bar 10μm. (d) Dot plot quantifying cilia length in WT RET and RET−/− pools. Bars represent the mean, **** p < 0.0001, ns not significant. (e) Dot plot quantifying the cilia number in WT RET and RET−/− pools. Each dot represents a field of view. Bars represent the mean, ns not significant [file 12860_2025_551_MOESM3_ESM.jpg]
