# Supplementary material for: Rescue of ciliogenesis and hyperglutamylation mutant phenotype in AGBL5−/− cell model of retinitis pigmentosa
Source: BMC Mol Cell Biol. 2025 Sep 9;26:27. doi: 10.1186/s12860-025-00551-x (PMC12418683; doi:10.1186/s12860-025-00551-x)
Supplement: Supplementary file 4 — Supplementary Tables S1–S6. Supplementary Table S1: Primer sequences used for PCR, RT-PCR, qPCR and sequencing. Supplementary Table S2: Primary antibodies used in western blot and fluorescent ICC. Supplementary Table S3: Secondary antibodies used in western blot and fluorescent ICC. Supplementary Table S4: AGBL5 transcript expression as transcripts per million reads (TPM) in WT and AGBL5-/- cells. Supplementary Table S5: TTLL gene expression as transcripts per million reads (TPM) in WT and AGBL5-/- A7 and A9 clones. Supplementary Table S6. AGBL5 protein interactants reported in the literature and references [file 12860_2025_551_MOESM4_ESM.pdf]

Table S1. Primer sequences used for PCR, RT-PCR, qPCR and sequencing

Use	Primer Name	Sequence 5’-3’
**Sequencing of *AGBL5* KO region**	AGBL5-F	GTCCCTCCTTTCTCCTTGCC
	AGBL5-R	AGGGGCTCATCTTGGACTCT
**Amplification of *AGBL5* transcript variants**	cDNA-AGBL5-F	GAGAGGGAGCGGGATCAC
	cDNA-AGBL5-NDM-F	TTCTTGTTTCCTGGCAGAGC
	cDNA-AGBL5-long and NDM-R	CTGTCACATGGAATCAGCGG
	cDNA-AGBL5-short-R	GGGCCAACAGTATATCTTCAAGC
**Sequencing of the *AGBL5* insert in the AGBL5-eGFP expression construct**	pcDNA-AGBL5-F1	GGGGATTGCTGTTCAGTTCTC
	pcDNA-AGBL5-F2	CATGAACAAGCAGAGCAAGC
	pcDNA-AGBL5-F3	CCCCGTCTAGAGCAGCTATT
	pcDNA-AGBL5-F4	CAGTTCCTGTCTCCCTCCTG
	pcDNA-AGBL5-F5	ATGTATGCCCGAGACCGTAG
	pcDNA-AGBL5-F6	CCTGTCTCCTGCTCCGAAAA
	pcDNA-AGBL5-F7	TGCTCACTCTTGTCCTCTGG
	pcDNA-AGBL5-R	CATAGTCAGGGGAAGAGGCA
	pcDNA-eGFP-R	GCTGAACTTGTGGCCGTTTA
**Sequencing of CRISPR *RET* and *TTLL5* KO regions**	RET_KO_F	TGTATGGTTCAGGTGCCCTT
	RET_KO_R	AGCCTCACTTAACCCCTGAC
	TTLL5_KO_F	GGCTGGAGGTGATTGGGA
	TTLL5_KO_R	GGAGAGAAGGGACATTCAAGC
**qPCRs performed in siRNA experiments**	GAPDH_QPCR_EX8_F	GTCTCCTCTGACTTCAACAGCG
	GAPDH_QPCR_EX9_R	ACCACCCTGTTGCTGTAGCCAA
	TTLL5_QPCR_EX7_F	GGACCGGGGACCTTGGATA
	TTLL5_QPCR_EX8_R	AATGTAACGGGAGACCAAAATGT
	ACTB_QPCR_EX4_F	CACTCTTCCAGCCTTCCTTC
	ACTB_QPCR_EX5_R	GTACAGGTCTTTGCGGATGT

Table S2. Primary antibodies used in western blot and fluorescent ICC

Target	Host	Class	Supplier	Cat No	Western Blot	ICC
**AAT Acetylated α-tubulin**	Mouse	Monoclonal	Sigma	T7451 Clone 6-11B-1	1:15,000	1:100
**AGBL5**	Rabbit	Polyclonal	Novus Bio	NBP3-10627	1:500	1:10 – 1:50
**ARL13B**	Rabbit	Polyclonal	Proteintech	17711-1-AP	–	1:500
**β-actin**	Mouse	Monoclonal	Sigma	A1978 Clone AC-15	1:5,000	–
**GAPDH**	Mouse	Monoclonal	Proteintech	60004-1-Ig	1:20,000	–
**GFP**	Mouse	Monoclonal	Proteintech	66002-1-Ig Clone 1E10H7	1:5,000	–
**GT335 glutamylated tubulin**	Mouse	Monoclonal	Adipogen	AG-20B-0020	1:5,000	1:500
**PolyE anti-polyglutamate chain**	Rabbit	Polyclonal	Adipogen	AG-25B-0030-C050	1:5,000	1:5,000
**Phospho-RET (Tyr905)**	Rabbit	Polyclonal	Cell Signalling	3221	1:1,000	–
**RET (C31B4)**	Rabbit	Monoclonal	Cell Signalling	3223	1:1,000	–
**Sec8 (EXOC4)**	Rabbit	Polyclonal	Proteintech	11913-1-AP	1:1000	–
**TTLL5**	Rabbit	Polyclonal	Abcam	Ab 187697	1:5,000	–
**USP9X**	Mouse	Monoclonal	Proteintech	68460-1-Ig Clone 1H6G11	1:5000	–
**USP9X**	Rabbit	Polyclonal	Proteintech	55054-1-AP	1:5000	–

Table S3. Secondary antibodies used in western blot and fluorescent ICC

Name	Host	Reactivity	Supplier	Cat No	Application	Dilution
**Goat anti-Rabbit IgG (H+L) Cross-Adsorbed, Alexa Fluor™ 488**	Goat	Rabbit	Thermo Fisher	A-11008	ICC	1:1,000
**Goat anti-Mouse IgG (H+L) Cross-Adsorbed, Alexa Fluor™ 568**	Goat	Mouse	Thermo Fisher	A-11004	ICC	1:1,000
**Donkey anti-Mouse IgG (H+L) Highly Cross-Adsorbed, Alexa Fluor™ 488**	Donkey	Mouse	Thermo Fisher	A-21202	ICC	1:1,000
**Donkey anti-Rabbit IgG (H+L) Highly Cross-Adsorbed, AlexaFluor™ 568**	Donkey	Rabbit	Thermo Fisher	A-10042	ICC	1:1,000
**Goat anti-rabbit immunoglobulins/HRP**	Goat	Rabbit	Agilent Dako	P0448	Chemi-luminescent Western Blot	1:5,000
**Goat anti-mouse immunoglobulins/HRP**	Goat	Mouse	Agilent Dako	P0447	Chemi-luminescent Western Blot	1:5,000

Table S4. *AGBL5* transcript expression as transcripts per million reads (TPM) in WT and *AGBL5*$^{-/-}$ A7 and A9 clones.

AGBL5 **Transcript**		**WT P14**	**WT P15**	**A7 P19**	**A7 P20**	**A9 P19**	**A9 P20**	**A9 P21**
**Ensembl Transcript ID**	**RefSeq Accession**	**TPM**	**TPM**	**TPM**	**TPM**	**TPM**	**TPM**	**TPM**
**ENST00000323064.12 AGBL5-201 (Short - 11 exons)**	NM_001035507.3	25.64	27.97	5.71	6.15	5.18	5.26	6.13
**ENST00000360131.5 AGBL5-202 (Canonical - 15 exons)**	NM_021831.6	3.62	4.45	2.37	2.40	2.05	2.16	2.31
**ENST00000421915.5 AGBL5-203**	-	0.62	1.46	1.36	0.86	1.10	1.05	0.95
**ENST00000437006.1 AGBL5-204**	-	0.54	0.38	0.97	0.54	0.58	0.55	0.72
**ENST00000441931.1 AGBL5-205**	-	4.69	3.98	4.70	3.70	4.39	2.31	2.92
**ENST00000451003.5 AGBL5-206**	-	1.35	0.89	0.84	0.50	1.22	0.97	0.66
**ENST00000453161.5 AGBL5-207**	-	0.00	0.40	0.13	0.00	0.00	0.00	0.00
**ENST00000477136.5 AGBL5-208**	-	0.20	0.71	0.26	0.08	0.26	0.20	0.21
**ENST00000487078.5 AGBL5-209 (NMD)**	-	3.66	3.06	2.55	1.77	2.29	2.15	2.55
**ENST00000489683.5 AGBL5-210**	-	1.93	3.08	1.06	1.31	0.84	0.84	0.81

Table S5. *TTLL* gene expression as transcripts per million reads (TPM) in WT and *AGBL5*$^{-/-}$ A7 and A9 clones

ENSG	Gene Symbol	WT P14	WT P15	A7 P19	A7 P20	A9 P19	A9 P20	A9 P21
ENSG00000100271	TTLL1	4.40	5.19	3.90	4.25	3.49	2.96	3.61
ENSG00000135912	TTLL4	12.89	12.08	15.22	13.92	14.69	14.85	13.98
ENSG00000100304	TTLL12	66.37	74.57	57.32	66.31	55.66	67.28	58.20
ENSG00000141543	TTLL3	38.32	51.01	49.12	57.18	48.89	50.43	45.12
ENSG00000148481	TTLL11	21.00	20.57	19.87	20.07	19.24	17.79	19.54
ENSG00000115128	TTLL7	37.72	34.87	33.33	33.57	37.39	33.63	34.31
ENSG00000065154	TTLL5	77.67	78.83	78.34	83.27	82.57	83.02	81.36

Table S6. AGBL5 protein interactants reported in the literature.

**Interactants**	**Detection Method**	**Cells/Tissue**	**Reference**
E6	Yeast two-hybrid pooling	*Saccharomyces cerevisiae*, human papillomavirus type 32	(1)
DLG4	Peptide array	Mouse Hippocampal or spinal cord samples	(2)
S	Proximity label	Human HEK293T and SARS-CoV-2	(3)
CP110	Anti-tag co-IP	Human HEK293T	(4)
GFOD1*, HAVCR2*, IL20RA, RELL2*, and TPCN2*	Anti-tag co-IP	Human HEK293T	(5)
FBRS, PCGF3, PCGF5, RYBP and TUBG1	Affinity capture	Human HEK293T	(6)
APPBP2, BTNL9, CAMK2A, DNAJA2, EFNB1, FTL, GFOD1*, HAVCR2*, HSPA8, KCNE3, PIPSL, PRPS2, RELL2*, SLC31A1 and TPCN2*	Affinity capture	Human HEK293T	(7)

Protein identified in seven different studies, method used to detect the interaction, and cells/tissues where the interaction was observed. The star (*) represents proteins identified in two separate studies.

References

1. Neveu G, Cassonnet P, Vidalain PO, Rolloy C, Mendoza J, Jones L, et al. Comparative analysis of virus-host interactomes with a mammalian high-throughput protein complementation assay based on Gaussia princeps luciferase. Methods. 2012;58(4):349-59.
2. Arbuckle MI, Komiyama NH, Delaney A, Coba M, Garry EM, Rosie R, et al. The SH3 domain of postsynaptic density 95 mediates inflammatory pain through phosphatidylinositol-3-kinase recruitment. EMBO Rep. 2010;11(6):473-8.
3. Datta S, Chen DY, Tavares AH, Reyes-Robles T, Ryu KA, Khan N, et al. High-resolution photocatalytic mapping of SARS-CoV-2 spike interactions on the cell surface. Cell Chem Biol. 2023;30(10):1313-22 e7.
4. Wang Y, Zhang Y, Guo X, Zheng Y, Zhang X, Feng S, et al. CCP5 and CCP6 retain CP110 and negatively regulate ciliogenesis. BMC Biol. 2023;21(1):124.
5. Huttlin EL, Bruckner RJ, Paulo JA, Cannon JR, Ting L, Baltier K, et al. Architecture of the human interactome defines protein communities and disease networks. Nature. 2017;545(7655):505-9.
6. Marcon E, Ni Z, Pu S, Turinsky AL, Trimble SS, Olsen JB, et al. Human-chromatin-related protein interactions identify a demethylase complex required for chromosome segregation. Cell Rep. 2014;8(1):297-310.
7. Huttlin EL, Bruckner RJ, Navarrete-Perea J, Cannon JR, Baltier K, Gebreab F, et al. Dual proteome-scale networks reveal cell-specific remodeling of the human interactome. Cell. 2021;184(11):3022-40 e28.
